# Empirical Failures of the Claim That Autistic People Lack a Theory of Mind

**DOI:** 10.1037/arc0000067

**Published:** 2019-12-09

**Authors:** Morton Ann Gernsbacher, Melanie Yergeau

**Affiliations:** University of Wisconsin—Madison; University of Michigan

**Keywords:** autism, theory of mind, convergent validity, predictive validity, reproducibility

## Abstract

The claim that autistic people lack a theory of mind—that they fail to understand that other people have a mind or that they themselves have a mind—pervades psychology. This article (a) reviews empirical evidence that fails to support the claim that autistic people are uniquely impaired, much less that all autistic people are universally impaired, on theory-of-mind tasks; (b) highlights original findings that have failed to replicate; (c) documents multiple instances in which the various theory-of-mind tasks fail to relate to each other and fail to account for autistic traits, social interaction, and empathy; (c) summarizes a large body of data, collected by researchers working outside the theory-of-mind rubric, that fails to support assertions made by researchers working inside the theory-of-mind rubric; and (d) concludes that the claim that autistic people lack a theory of mind is empirically questionable and societally harmful.

## SCIENTIFIC ABSTRACT

The assertion that autistic people lack a theory of mind—that they fail to understand that other people have a mind or that they themselves have a mind—pervades psychology. In this article, we critically examine the empirical basis of this assertion. We review empirical evidence that fails to support the claim that autistic people are uniquely impaired, much less that all autistic people are universally impaired, on theory-of-mind tasks. We highlight seminal theory-of-mind findings that have failed to replicate. We document multiple instances in which the various theory-of-mind tasks fail to converge and fail to predict autistic traits, social interaction, and empathy. We summarize a large body of data, collected by researchers working outside the theory-of-mind rubric, that fails to support assertions made by researchers working inside the theory-of-mind rubric. We conclude that the claim that autistic people lack a theory of mind is empirically questionable and societally harmful.

Most of us have a theory of mind in that we can guess what others are thinking and how that might differ from what we are thinking. Those with autism can be thought of as mindblind in that they cannot imagine what others might be thinking, or even that others are thinking. … To them, it would be like looking at the headlights of a car to determine why the car just did what it did, or what information it is trying to convey to us.

—The Encyclopedia of Neuropsychological Disorders

(Soper & Murray, 2012, p. 125)

The assertion that autistic^1^ people lack a theory of mind—that they fail to understand that other people have a mind or that they themselves have a mind—pervades psychology. The assertion is taught across a wide range of psychology textbooks (Coon, Mitterer, & Martini, 2018; Kellogg, 2007; Kirk, Gallagher, Coleman, & Anastasiow, 2008; Mash & Wolfe, 2015; Myers, 2009, 2012; Sigelman & Rider, 2017). The assertion is argued by psychologists in state and federal court cases (Carter v. Superintendent, 2011; New Jersey v. Burr, 2007; United States v. Geanakos, 2017). The assertion is promoted by thousands of psychology articles; in fact, the vast majority—over 75%—of the top 500 articles indexed by Google Scholar (for “theory of mind” and “autism”) simply assert that autistic people lack a theory of mind rather than provide original data to buttress the claim (Gernsbacher, 2018a).^2^ Clearly, the assertion that autistic people lack a theory of mind has become one of psychology’s sacred topics, a critical evaluation of which the current special issue solicited.

In this article, we review empirical evidence that fails to support the claim that autistic people are uniquely impaired, much less that all autistic people are universally impaired, on theory-of-mind tasks. We highlight seminal theory-of-mind findings that have failed to replicate. We document multiple instances in which various theory-of-mind tasks fail to converge and fail to predict autistic traits, social interaction, and empathy. We summarize a large body of data, collected by researchers working outside the theory-of-mind rubric, that fail to support assertions made by researchers working inside the theory-of-mind rubric. We conclude that the claim that autistic people lack a theory of mind is empirically questionable and societally harmful.

## Failures of Specificity

For nearly two decades, Simon Baron-Cohen and his colleagues claimed that poor performance on theory-of-mind tasks uniquely characterized autistic people (see Table 1).The initial claim was staked on autistic children’s performance on a theory-of-mind task called False Belief. In a False Belief task, a child might be introduced to two puppets, one named Sally and the other Anne. The child watches as the Sally puppet places a possession, such as a marble, inside a basket. Then, the Sally puppet is taken away, and the Anne puppet moves the marble from its previous location to another location, such as inside a box. When the Sally puppet is represented, the child is asked orally, “Where will Sally look for her marble?” If the child answers with the location where the marble actually is, rather than the location where the first puppet placed the marble, the child is considered to have failed the False Belief task and to lack a theory of mind.

Other tasks have been used to assess theory of mind; some of the more popular ones appear in Table 2. But it was autistic children’s performance on False Belief tasks that propelled Baron-Cohen and his colleagues’ claim that autistic people uniquely lack a theory of mind.

However, autistic children are not unique in failing False Belief tasks; so too do children with specific language impaiiment (Loukusa, Mäkinen, Kuusikko-Gauffin, Ebeling, & Moilanen, 2014; Norbury, 2005); Down syndrome (Zelazo, Burack, Benedetto, & Frye, 1996); Williams syndrome (van Herwegen, Dimitriou, & Rundblad, 2013); Prader Willi syndrome (Lo, Siemensma, Collin, & Hokken-Koelega, 2013); cerebral palsy (Caillies, Hody, & Calmus, 2012; Dahlgren, Dahlgren Sandberg, & Hjelmquist, 2003); Fragile X (Cornish et al., 2005); epilepsy (Raud, Kaldoja, & Kolk, 2015); and neurofibromatosis type I (Payne, Porter, Pride, & North, 2016), as well as children exposed prenatally to maternal smoking (Reidy, Ross, & Hunter, 2013) and drinking (Rasmussen, Wyper, & Talwar, 2009). Indeed, the more atypical the child, the more likely they are to fail false belief tasks.

Even typically developing children with fewer rather than more siblings (Jenkins & Astington, 1996; Peterson, 2000), with lower rather than higher socioeconomic status (Hughes & Ensor, 2005), or with fewer rather than more adult relatives living nearby (Lewis, Freeman, Kyriakidou, Maridaki-Kassotaki, & Berridge, 1996) are more likely to fail False Belief tasks, as are children who are blind (Brambring & Asbrock, 2010; Green, Pring, & Swettenham, 2004; Minter, Hobson, & Bishop, 1998; Peterson, Peterson, & Webb, 2000) or deaf/hard of hearing (Figueras-Costa & Harris, 2001; Jackson, 2001; Lundy, 2002; Meristo et al., 2007; Moeller & Schick, 2006; Peterson & Siegal, 1995; Russell et al., 1998).

More recently, Baron-Cohen has acknowledged that a lack of theory of mind “may not be specific” to autistic people (Baron-Cohen, 2009, p. 70; 2010, p. 169). For nearly 30 years, other researchers have also tried to correct this inaccurate claim (Eisenmajer & Prior, 1991; Frye, Zelazo, & Burack, 1998; Prior, Dahlstrom, & Squires, 1990; Tager-Flusberg, 2001, 2007; Yirmiya & Shulman, 1996; Yirmiya, Erel, Shaked, & Solomonica-Levi, 1998; Zelazo, Jacques, Burack, & Frye, 2002). But the erroneous claim that only autistic people, “together with robots and chimpanzees” lack a theory of mind (Pinker, 2002, p. 62; see also Mitchell, 1997) and are therefore “biologically set apart from the rest of humanity in lacking the basic machinery” (Baron-Cohen, 2009, p. 73) echoes throughout psychological literature, practice, and instruction (cf. Gernsbacher, 2007; Yergeau, 2013; Yergeau & Huebner, 2017).

## Failures of Universality

A lack of a theory of mind is often assumed to be not only a unique characteristic of autistic people, but also a universal characteristic of all autistic people. Repeatedly, Baron-Cohen has claimed that “mindblindness … is universal in applying to all individuals on the autistic spectrum” (Baron-Cohen, 2008a, p. 61; Baron-Cohen, 2008b, p. 113; Baron-Cohen, 2009, p. 70; Baron-Cohen, 2010, p. 169; Baron-Cohen, 2011a, p. 40; Baron-Cohen, 2011b, p. 629; see also Table 3). This assumed universality has been widely promoted across psychology, as the opening quote of our article illustrates. However, as other authors note, many autistic children and adults pass theory-of-mind tasks; therefore, these other authors rightly argue that “mindblindness” cannot be a universal characteristic of autism (e.g., Bailey, Phillips, & Rutter, 1996; Bauminger & Kasari, 1999; Beversdorf et al., 1998; Boucher, 2012; Buitelaar, van der Wees, Swaab-Barneveld, & van der Gaag, 1999b; Charman, 2000; Ozonoff, Rogers, & Pennington, 1991).

Why do some autistic participants pass theory-of-mind tasks while others do not? Numerous researchers have aptly noted that theory-of-mind tasks rely heavily on spoken language (see Gernsbacher & Frymiare, 2005, and Gernsbacher & Pripas-Kapit, 2012, for reviews). For example, nearly half the variance in participants’ performance on False Belief tasks can be predicted by their spoken language comprehension (Capage & Watson, 2001); nearly three fourths can be predicted by their facility with vocabulary (Steele, Joseph, & Tager-Flusberg, 2003) and appreciation of grammar (Peterson, Wellman, & Slaughter, 2012). In longitudinal studies, vocabulary predicts False Belief performance more powerfully than age (Steele et al., 2003); in studies comparing autistic to nonautistic participants, vocabulary predicts False Belief performance more powerfully than whether the participants are autistic (Loukusa et al., 2014; Norbury, 2005; see also Milligan, Astington, & Dack’s, 2007, meta-analysis with over 100 studies of typically developing children; Yirmiya, Erel, Shaked, and Solomonica-Levi [1998], meta-analysis with 40 studies of autistic children; and Gernsbacher, 2018a, for studies published after these meta-analyses).

Other theory-of-mind tasks also draw heavily on spoken language. Happé’s (1994a) Strange Stories task (see Table 2) requires comprehending complex stories and answering complex questions, which is why complex language comprehension can be the task’s “only” predictor (Shaked, Gamliel, & Yirmiya, 2006, p. 183), and vocabulary can account for more than three fourths of the variance (de Lima Velloso, Duarte, & Schwartzman, 2013; see also Abell & Hare, 2005; Botting & Conti-Ramsden, 2008; Dyck, Ferguson, & Shochet, 2001; Frölander et al., 2014; Kaland et al., 2005; Loth, Gómez, & Happé, 2008; Scheeren, de Rosnay, Koot, & Begeer, 2013; Solomon, Goodlin-Jones, & Anders, 2004).

Even performance on Baron-Cohen, Wheelwright, Hill, Raste, and Plumb’s (2001) Reading-the-Mind-in-the-Eyes task “involves sophisticated vocabulary” (Muller et al., 2010, p. 1095), which is why the best predictor of Reading-the-Mind-in-the-Eyes can be Speaking-Aloud-Hard-to-Pronounce-Words (Lawrence, Shaw, Baker, Baron-Cohen, & David, 2004) and why vocabulary and grammar can account for nearly half the variance (Bennett et al., 2013; see also Botting & Conti-Ramsden, 2008; Castelli et al., 2011; Dorris, Espie, Knott, & Salt, 2004; Olderbak et al., 2015; Pino et al., 2017; Peterson & Miller, 2012).

Because theory-of-mind tasks rely heavily on “fairly complex language” (San José Cáceres, Keren, Booth, & Happé, 2014, p. 608) and because autism, by diagnostic definition, involves communication impairment (Gernsbacher, Morson, & Grace, 2016), it is unsurprising that autistic participants with communication impairment perform less well than nonautistic participants without communication impairment. And because autistic people vary in their communication impairment (Gernsbacher, Geye, & Ellis Weismer, 2005), it is unsurprising that autistic people vary in their theory-of-mind task performance.

The heavy reliance of theory-of-mind tasks on language has led theory-of-mind proponents to claim that autistic people who pass theory-of-mind tasks must be using their linguistic abilities to “hack out” the answers (Happé, 1995, p. 853; Tager-Flusberg, 2001, p. 185). This claim might seem superficially sound, but it is hard to reconcile with the fact that autistic people, on average, have communication impairments. How and why would autistic people preferentially use language to “hack out” the answers while nonautistic people, without communication impairments, do not? A related claim made by those who assume that all autistic people must lack a theory of mind, is that autistic people who pass theory-of-mind tasks must use some unknown “logic” or post hoc “strategy” (Baron-Cohen, 2006, p. 868; Frith, Happé, & Siddons, 1994, p. 110; Happé, 1994a, p. 130, 1994b, p. 220). But such post hoc claims seem to fail their own test of logic.^3^

## Failures of Replication

Reproducibility is the cornerstone of science, as psychology’s current focus on replication illustrates (Gernsbacher, 2018b, 2018c, 2018d; Spellman, 2015; Tackett et al., 2017). However, when tests of reproducibility are applied to claims about autism and theory of mind, the seminal findings frequently fail.

For example, cognizant of the heavy reliance on language by most theory-of-mind tasks, Baron-Cohen, Leslie, and Frith (1986) designed a nonverbal task. Children were given a scrambled set of four pictures and told to arrange the pictures in a coherent order. One set of pictures displayed a boy standing at the top of a hill with a basketball-sized rock next to his foot; another picture displayed the boy with his foot close to the rock, as though ready to kick it; another picture displayed the rock halfway down the hill; and another picture displayed the rock at the bottom of the hill. Baron-Cohen et al. (1986) deemed this type of picture sequence “mechanical,” and autistic children were almost perfect in sequencing such pictures. Oddly, typically developing children performed below 50% correct on these “mechanical” pictures—which most likely was unexpected because Baron-Cohen et al. (1986, p. 116) deemed these “mechanical” pictures “the simplest.”

Another set of pictures displayed a boy sitting on the ground holding an ice cream cone to his mouth with a girl standing nearby; in another picture, the ice-cream-holding boy is looking at the girl who, in this picture, is also sitting on the ground; in another, the girl is reaching for the boy’s ice cream cone while he stretches his arm as far as possible away from the girl’s reach; in the final picture, the girl holds the ice cream cone to her mouth, while the boy rubs his eyes. Autistic and typically developing children were equally adept at arranging this type of picture sequence, which Baron-Cohen et al. (1986, p. 115) deemed “behavioral” and, quite curiously, not an assay of the characters’ intentions or requiring an understanding of “mental states.”

An example of the last type of picture sequence displayed a girl holding a teddy bear in her arms, while a flower extends from the ground beside her; in another picture, the girl is turned completely to one side and is holding the flower’s stem, while the teddy bear is on the ground behind her; in another, the girl is holding the flower to her nose, while a boy, standing behind the girl, reaches for the teddy bear on the ground; in the final picture, the girl is turned around, there’s no boy or teddy bear, and the girl’s mouth is wide open. Baron-Cohen et al. (1986, p. 116, 224) deemed this picture sequence “intentional,” and the typically developing children, who performed so shockingly poorly on the “simplest” mechanical pictures performed nearly perfectly on these pictures, whereas the autistic children performed poorly. Baron-Cohen et al. (1986, p. 113) used these data to claim that “a specific cognitive deficit … prevents the development of a ‘theory of mind’ in the autistic child.”

Four research teams, of whom we are aware, have published attempts to directly replicate these results—and none could do so. Using the same stimuli, procedures, and analyses, no other research team has replicated the finding that autistic participants perform significantly worse than typically developing participants on the “intentional” picture sequences (“there were no group differences on the intentional subtest of the picture sequencing measure,” Ozonoff, Pennington, & Rogers, 1991, p. 1093; “contrary to … previous findings (Baron-Cohen et al., 1985, 1986), [the intentional condition of the Picture Sequence Task] … failed to reveal significant differences,” Oswald & Ollendick, 1989, p. 122; “no two groups were significantly different [on the Intentional picture sequence],” Buitelaar, van der Wees, Swaab-Barneveld, & van der Gaag, 1999a, p. 46; “The [autistic] participants were close to ceiling … on the intentional Picture Sequencing items,” Brent, Rios, Happé, & Charman, 2004, p. 286).

Not only does Baron-Cohen et al.’s (1986) seminal theory-of-mind study fail to replicate, but its initially reported effect size, *d* = −1.714, looms unusually large (Ioannidis, 2008). In contrast, its replications’ pooled effect size is normatively tiny, *d* = −0.039 (Gernsbacher, 2018a), with a confidence interval (CI) that easily overlaps zero (i.e., 99.9% CI [−0.690, 0.611], giving us 99.9% confidence that the true effect includes zero). We are also unaware of any published studies that have replicated Baron-Cohen et al.’s (1986, pp. 116, 224) report that typically developing participants are dramatically worse on the “simplest” mechanical picture sequences than on the “fairly difficult” intentional picture sequences (cf. Rhys-Jones & Ellis, 2000; Savina & Beninger, 2007).

Similarly, Baron-Cohen, Leslie, and Frith (1985)’s seminal study reporting that autistic participants are prone to fail first-order False Belief tasks (see Table 2) is also prone to fail replication (e.g., “No statistically significant difference between groups were found in the test of first-order theory of mind … These findings suggest that the theory of mind model has its limitations in explaining autism,” Dahlgren & Trillingsgaard, 1996, pp. 761, 759; “the children with autism did not underperform on this task,” Russell & Hill, 2001, p. 236; “Contrary to … previous findings (Baron-Cohen et al., 1985) … [the replication] failed to reveal significant differences,” Oswald & Ollendick, 1989, p. 122; “these were not statistically significant differences,” Fitzpatrick, Diorio, Richardson, & Schmidt, 2013, p. 7; “no differences emerged,” Yirmiya & Shulman, 1996, p. 2045; “[the replication’s] findings … are not consistent with … previous reports,” Yirmiya, Solomonica-Levi, Shulman, & Pilowsky, 1996, p. 1011; see also Moran et al., 2011).

Likewise, Baron-Cohen’s (1989b) report that autistic participants are prone to fail second-order False Belief tasks (see Table 2) is also prone to fail replication (e.g., “No group differences were found in performance on the control or test questions,” Tager-Flusberg & Sullivan, 1994, p. 577; “was no difference between normal and autistic children’s performance,” Leekam & Prior, 1994, p. 907; “no significant association between group membership and proportion of items passed,” Bowler, 1992, p. 885; “our findings are inconsistent with early studies of False Belief abilities in autism,” Bauminger & Kasari, 1999, p. 85; “The present findings contradict the claims of proponents of … the theory of mind … hypothesis of autism,” Buitelaar et al., 1999a, p. 53).

Furthermore, Happé’s (1994a) report that autistic participants who pass first- or second-order False Belief tasks nonetheless fail an “advanced test of theory of mind” (Strange Stories) has also failed at replication (e.g., “counter to our expectations, no group differences were found on any of the stories,” Scheeren et al., 2013, p. 632; “no group differences in … the Strange Stories,” Senju, Southgate, White, & Frith, 2009, p. 884; “In line with prior findings by Senju et al. (2009), no performance differences … were observed in the [Strange Stories task],” Schuwerk, Vuori, & Sodian, 2015, p. 466; see also Gillott, Furniss, & Walter, 2004; Murray et al., 2017; Ponnet, Roeyers, Buysse, De Clercq, & Van Der Heyden, 2004; Roeyers, Buysse, Ponnet, & Pichal, 2001; Schneider, Slaughter, Bayliss, & Dux, 2013; Spek, Scholte, & van Berckelaer-Onnes, 2010; White, Hill, Happé, & Frith, 2009; Wilson et al., 2014). In fact, the pooled effect size of over a dozen systematically reviewed direct replications (Gernsbacher, 2018a) not only overlaps zero (*d* = −0.229, 99.9% CI [−0.479, 0.021]), but also fails to overlap the pooled effect size of the seminal studies (*d* = −1.696, 99.9% CI [−0.932, −2.460]).

Perhaps the failure of these seminal studies to replicate derives from their small sample sizes. Samples two to three times larger are needed to reliably test the somewhat obvious hypothesis that people who like spicy food are more likely to report liking Indian food or that people who like eggs are more likely to report eating egg salad (Simmons, Nelson, & Simonsohn, 2013). Even reliably testing the hypothesis that men weigh more than women requires samples more than thrice the size of those collected in many of Baron-Cohen’s seminal theory-of-mind studies (e.g., autistic participants *N* = 10, Baron-Cohen, 1989b; *N* = 15, Baron-Cohen, 1992; Baron-Cohen et al., 2001; *N* = 16, Baron-Cohen, Jolliffe, Mortimore, & Robertson, 1997; Baron-Cohen, Wheelwright, & Jolliffe, 1997; *N* = 17, Baron-Cohen, 1991c; *N* = 20, Baron-Cohen et al., 1985, 1995; *N* = 21, Baron-Cohen et al., 1986).

Despite these seminal studies’ precariously small sample sizes and their lack of replication, their grander claims continue to rebound through textbooks and scholarly literature, within and outside of psychology, and they ricochet through public vernacular. The robustness of these claims, if not the robustness of their supporting evidence, could well have deterred other researchers from publishing conflicting results (Franco, Malhotra, & Simonovits, 2014).

## Failures of Convergent Validity

Several tasks have been proposed to assess theory of mind, as Table 2 illustrates. However, in more recent studies, many with quite large samples of autistic and nonautistic participants, these tasks fail to converge. These repeated failures of convergence seriously question the tasks’ validity.

For example, performance on Happé’s (1994a) Strange Stories task fails to correlate significantly with performance on Baron-Cohen et al.’s (2001) Reading-the-Mind-in-the-Eyes task (*N* = 123 nonautistic adults, Ahmed & Miller, 2011; *N* = 100 autistic children, Lukito et al., 2017; *N* = 90 autistic adolescents, Hollocks et al., 2014; *N* = 89 autistic and 89 nonautistic adults, Wilson et al., 2014; *N* = 61 autistic and 32 nonautistic adults, Spek et al., 2010; *N* = 60 nonautistic adolescents and 60 nonautistic adults, Vetter, Leipold, Kliegel, Phillips, & Altgassen, 2013; *N* = 53 nonautistic adults, Chen et al., 2017; *N* = 50 nonautistic adults, Scherzer, Leveillé, Achim, Boisseau, & Stip, 2012; see also Adler, Nadler, Eviatar, & Shamay-Tsoory, 2010; Brent et al., 2004; Dziobek et al., 2006; Farrant et al., 2005; Kaland, Callesen, Møller-Nielsen, Mortensen, & Smith, 2008; Kristen, Rossmann, & Sodian, 2014; Roeyers et al., 2001).^4^ In fact, the average correlation between performance on the Strange Stories task and the Reading-the-Mind-in-the-Eyes task, weighted across 27 systematically reviewed samples (Gernsbacher, 2018a), is only 0.089, with a CI that overlaps zero (i.e., 99.9% CI [ −.001, .178]).^5^

Similarly, the Strange Stories task fails to correlate significantly with the Animated Triangles task (*N* = 100 autistic children, Lukito et al., 2017; *N* = 90 autistic adolescents, Hollocks et al., 2014; *N* = 89 autistic and 89 nonautistic adults, Wilson et al., 2014; *N* = 80 nonautistic adults, Brewer, Young, & Barnett, 2017; see also Clemmensen et al., 2016). The Strange Stories task also fails to correlate significantly with the Faux Pas task (*N* = 123 nonautistic adults, Ahmed & Miller, 2011; *N* = 61 autistic and 32 nonautistic participants, Spek et al., 2010), particularly when language comprehension is controlled.

Reading-the-Mind-in-the-Eyes fails to correlate significantly with (a) the Faux Pas task (*N* = 123 nonautistic adults, Ahmed & Miller, 2011; *N* = 80 nonautistic adults, Li et al., 2013; *N* = 70 nonautistic adults, Duval, Piolino, Bejanin, Eustache, & Desgranges, 2011; *N* = 61 autistic and 32 nonautistic adults, Spek et al., 2010; *N* = 53 nonautistic adults, Chen et al., 2017; *N* = 50 nonautistic adults, Scherzer et al., 2012), (b) the Animated Triangles task (*N* = 70 nonautistic adults, Duval et al., 2011; White, Coniston, Rogers, & Frith, 2011), (c) False Belief task (*N* = 100 autistic participants, Lukito et al., 2017; *N* = 90 autistic adolescents, Hollocks et al., 2014; see also Ozonoff et al., 1991), and (d) with other theory-of-mind tasks (e.g., the Hinting task, *N* = 134 nonautistic adults, Gooding & Pflum, 2011; *N* = 73 nonautistic adults, Bora et al., 2005; *N* = 50 nonautistic adults, Scherzer et al., 2012).

Even False Belief tasks can fail to correlate significantly with each other (e.g., Charman & Campbell, 1997; Duval et al., 2011; Hughes, 1998). The lack of convergent validity among theory-of-mind tasks undermines the core construct validity of theory of mind.

## Failures of Predictive Validity

If theory-of-mind tasks assay “the basic machinery for social engagement” (Baron-Cohen, 2009, p. 73), then performance on theory-of-mind tasks should predict socioemotional function. But numerous studies document failures of prediction. For example, performance on theory-of-mind tasks fails to significantly predict

- *autistic traits in either autistic or nonautistic participants*, as measured by clinicians’ observation, self-report, or informant-report (*N* = 1513 nonautistic adults, Kunihira, Senju, Dairoku, Wakabayashi, & Hasegawa, 2006; *N* = 638 nonautistic children, Ronald, Viding, Happé, & Plomin, 2006; *N* = 395 autistic adults, Lombardo et al., 2015; *N* = 220 nonautistic adults, Ragsdale & Foley, 2011; *N* = 206 nonautistic men, Voracek & Dressler, 2006; *N* = 194 autistic and 60 nonautistic children, Scheeren et al., 2013; *N* = 178 autistic men, 168 nonautistic women, and 152 nonautistic men, Baron-Cohen et al., 2015; *N* = 108 nonautistic adults, Melchers, Montag, Markett, & Reuter, 2015; *N* = 100 autistic adolescents, Lukito et al., 2017; *N* = 100 autistic adolescents, Jones et al., 2018; *N* = 89 autistic and 89 nonautistic adults, Wilson et al., 2014; *N* = 79 nonautistic women, Valla et al., 2010; *N* = 56 autistic children, Salter, Seigal, Claxton, Lawrence, & Skuse, 2008; see similar results with smaller samples in Bryant, Coffey, Povinelli, & Pruett, 2013; Burnside, Wright, & Poulin-Dubois, 2017; Clemmensen et al., 2016; Dziobek et al., 2006; Murray et al., 2017; Ozonoff & McEvoy, 1994)
- *empathy and emotional understanding* (*N* = 484 nonautistic adults, Olderbak et al., 2015; *N* = 395 autistic adults, Lombardo et al., 2015; *N* = 342 nonautistic adolescents, Sharp & Vanwoerden, 2014; *N* = 220 nonautistic adults, Ragsdale & Foley, 2011; *N* = 200 nonautistic adults, Vellante et al., 2013; *N* = 178 autistic men, 168 nonautistic women, and 152 nonautistic men, Baron-Cohen et al., 2015; *N* = 162 nonautistic adults, Ferguson & Austin, 2010; *N* = 121 nonautistic adolescents and adults, Gökçen, Frederickson, & Petrides, 2016; *N* = 108 nonautistic adults, Melchers et al., 2015; *N* = 89 autistic and 89 nonautistic adults, Wilson et al., 2014; *N* = 58 nonautistic children, Tsang, Gillespie-Lynch, & Hutman, 2016; *N* = 53 nonautistic adults, Lawrence et al., 2004; see similar results with smaller samples in Carroll & Chiew, 2006; Campbell et al., 2011; Muller et al., 2010; Peterson, 2014)
- *everyday social skills* (*N* = 398 nonautistic children, Shahrivar, Tehrani-Doost, Khorrami Banaraki, Mohammadzadeh, & Happé, 2017; *N* = 164 and 140 nonautistic adults, Ames & Kammrath, 2004; *N* = 124 nonautistic adolescents, Botting & Conti-Ramsden, 2008; *N* = 101 nonautistic children, Lunn, Lewis, & Sherlock, 2015; *N* = 97 nonautistic children, Lew et al., 2015; *N* = 65 nonautistic children, Raud et al., 2015; *N* = 63 nonautistic adults, Stanford, Messinger, Malaspina, & Corcoran, 2011; *N* = 60 nonautistic children, Raud et al., 2015; *N* = 53 nonautistic adults, Chen et al., 2017; *N* = 50 nonautistic adults, McCleery et al., 2012; see similar results with smaller samples of autistic children and adults in Bennett et al., 2013; Fombonne, Siddons, Achard, Frith, & Happé, 1994; Frith et al., 1994^6^; Hughes, Soares-Boucaud, Hochmann, & Frith, 1997; Joseph & Tager-Flusberg, 2004; Prior et al., 1990; Shaked et al., 2006; Sparrevohn & Howie, 1995; and smaller samples of nonautistic children and adults in Carroll & Chiew, 2006; Tso, Grove, & Taylor, 2010; Watson, Nixon, Wilson, & Capage, 1999; Clegg, Hollis, Mawhood, & Rutter, 2005)
- *social attention, cooperation, anticipation, persuasion, deception, and avoidance* (*N* = 402 nonautistic adolescents, Hünefeldt, Laghi, Ortu, & Belardinelli, 2013; *N* = 77 autistic children, Angus, de Rosnay, Lunenburg, Meerum Terwogt, & Begeer, 2015; see similar results with smaller samples of autistic children in Burnside et al., 2017; Chin & Bernard-Opitz, 2000; Kristen, Vuori, & Sodian, 2015; Peterson, Slaughter, & Wellman, 2018; and smaller samples of nonautistic children in Brooks & Meltzoff, 2015; Ding, Wellman, Wang, Fu, & Lee, 2015);
- *peer relations and pro-social behavior* (*N* = 263 nonautistic children, Botting & Conti-Ramsden, 2008; *N* = 128 nonautistic children, Bosacki & Astington, 1999; *N* = 115 nonautistic children, Badenes, Clemente Estevan, & García Bacete, 2000; *N* = 115 nonautistic girls and *N* = 115 nonautistic boys, Devine & Hughes, 2013; *N* = 51 nonautistic children, Capage & Watson, 2001; see similar results with smaller samples of autistic and nonautistic children, adolescents, and adults in Begeer, Malle, Nieuwland, & Keysar, 2010; Campbell et al., 2011; Lalonde & Chandler, 1995; Travis, Sigman, & Ruskin, 2001).

Indeed, when Baron-Cohen and his colleagues applied machine learning to categorize a large sample (*N* = 395) of autistic adults into those who perform better versus worse on a theory-of-mind task, the researchers were unable to identify any variable that patterned with theory-of-mind performance “including sex/gender, age, depression or anxiety symptoms, autistic traits, trait empathy, and autism symptom severity” (Lombardo et al., 2015, p. 2). The only characteristic that reliably patterned with theory-of-mind performance was language dexterity.

Finally, if theory-of-mind tasks truly assay the ability to infer other people’s “intentions, goals and desires” (Baron-Cohen et al., 1995, p. 381), and if autistic people lack a theory of mind, then autistic people should fare poorly at inferring other people’s intentions, goals, and desires. But, as Table 4 illustrates, autistic people of all ages skillfully understand other persons’ intentions, goals, and desires. This large body of data, collected by researchers working outside the theory-of-mind rubric, demonstrates another failure of the claim that autistic people lack a theory of mind.

## Conclusion and Recommendation

In this article, we have demonstrated how the claim that autistic people lack a theory of mind fails empirically; it fails in its specificity, universality, replicability, convergent validity, and predictive validity. Despite these numerous empirical failures, the claim pervades psychology and well beyond. It is embraced by scholars in philosophy (Barnbaum, 2008), sociology (Willey, Subramaniam, Hamilton, & Couperus, 2015), economics (Singer & Fehr, 2005), anthropology (Boyer, 2000), robotics (Scassellati, 2002), and narratology (Barnes, 2012; Goodman, 2010: Zunshine, 2008). It colors contemporary entertainment (e.g., *The Good Doctor*, Yegorova, 2017), and it headlines informational websites (Autism Society of Indiana, n.d.; Autism Society of Minnesota, 2016; Autism Speaks, 2018; Scottish Autism, n.d.; Seattle Children’s Hospital, 2016). It has spawned unusual speculations, evoking metaphysical (Suddendorf & Corballis, 1997), psychoanalytic (Mayes, Cohen, & Klin, 1993), and neurochemical (Abu-Akel, 2003; Abu-Akel & Shamay-Tsoory, 2011) explanations.

The claim that autistic people lack a theory of mind is so entrenched that when existing measures fail to support the claim, researchers create new measures. For example, Baron-Cohen and his colleagues motivated the need for a new theory-of-mind task by claiming that autistic adults must “have a selective theory of mind … deficit,” even though existing theory-of-mind tests “are not subtle enough to detect [that] deficit” (Rutherford, Baron-Cohen, & Wheelwright, 2002, p. 189). Rajendran and Mitchell (2007) suggest, as do we, that “the development of advanced tests [is] a post hoc response in finding data anomalous to the theory of mind hypothesis” (p. 229; i.e., data that do not support the claim that autistic people lack a theory of mind).

The development of more and more theory-of-mind tests resembles a methodological arms race. The deployment of first-order False Belief tasks escalates to second-order False Belief tasks, which escalate to the so-called advanced theory-of-mind tasks (Strange Stories, Reading-the-Mind-in-the-Eyes, and Animated Triangles) and then to the Strange Stories Film task (Murray et al., 2017), the Comic Strip task (Sivaratnam, Cornish, Gray, Howlin, & Rinehart, 2012), and the Beauty Contest task (Pantelis & Kennedy, 2017)—all in pursuit of finding a task to support the claim that autistic people lack a theory of mind, when previous tasks fail to support the claim.

Most recently, “implicit” theory-of-mind tasks have been developed (Schneider et al., 2013; Schuwerk et al., 2015; Senju et al., 2009; but see Schuwerk, Priewasser, Sodian, & Perner, 2018, and Kulke, von Duhn, Schneider, & Rakoczy, 2018, for difficulties replicating measures of implicit theory of mind). As Rajendran and Mitchell (2007) note, researchers and their deployment of increasingly “advanced tests have turned … logic on its head.” The drive to create more and more theory-of-mind tasks “seem to be premised on the assumption” that autistic people lack a theory of mind; therefore, “tests which do not reveal this must be insensitive or unsuitable” (p. 229).

There has even been a move toward asking nonautistic parents to gauge their autistic offspring’s theory of mind (Hutchins, Prelock, & Bonazinga, 2012), which is problematic for at least two reasons. First, as autistic scholars have explained (e.g., Sinclair, 1993) and as empirical data demonstrate (e.g., Gernsbacher, Stevenson, & Dern, 2017), nonautistic people are often as disadvantaged when trying to understand autistic people as vice versa. Milton (2012) refers to this dilemma as the “double empathy problem” (see also Gernsbacher, 2006), which Loftis (2015, p. 10) illustrates with the following conundrum: “If autistics truly have a deficit in [theory of mind], then why is it that neurotypicals find it so difficult to intuit the intentions of autistic people”?

Second, most everyone misjudges their own theory-of-mind performance (Ames & Kammrath, 2004; Realo et al., 2003; Zaki, Bolger, & Ochsner, 2008). For example, an improbable eight out of 10 U.S. college students rate their own theory-of-mind ability as better-than-average (in contrast, a more probable half rate as more logically average their public speaking ability, social self-confidence, computer skills, physical health, emotional health, creativity, and propensity for risk taking, Higher Education Research Institute, 2017). Thus, it is unlikely that nonautistic parents can accurately assess their own, let alone their autistic offspring’s, theory-of-mind abilities. As even the creators of a child’s version of Reading-the-Mind-in-the-Eyes task admit, “it is unknown what the child [in the stimulus photographs] was actually feeling” because the stimulus photographs “were all derived from naturalistic settings (e.g., taken by parents) rather than being posed specifically for an experiment” (Pino et al., 2017, p. 2746).

Some researchers willingly admit that we do not know what theory of mind is (Schaafsma, Pfaff, Spunt, & Adolphs, 2015), much less how to measure it. Despite this uncertainty, other researchers claim with certainty that “autism is a clear illustration of what human life would be like if one lacked a theory of mind” (Baron-Cohen, 2000a, p. 266).

For example, philosopher David Livingstone Smith (2007, p. 172) claims that autistic people “live in a world in which nothing has a mind” and “perceive [other] people as hunks of flesh moving mindlessly through space.” Developmental psychologist Alison Gopnik ventures even further, graphically describing how she envisions autistic people perceive other people:

Around me bags of skin are draped over chairs, and stuffed into pieces of cloth, they shift and protrude in unexpected ways. … Two dark spots near the top of them swivel restlessly back and forth. A hole beneath the spots fills with food and from it comes a stream of noises. Imagine that the noisy skin-bags suddenly moved toward you, and their noises grew loud, and you had no idea why, no way of explaining them or predicting what they would do next. (Gopnik as quoted in Baron-Cohen, 1995, pp. 4–5; Gerrans, 2002, pp. 312–313; and Smith, 2007, p. 172)

Along with the stigma promulgated by such renditions, the claim that autistic people lack a theory of mind causes societal harm (Dinishak & Akhtar, 2013). Because a lack of theory of mind is believed to impair autistic people’s understanding of their selves, in addition to their understanding of others, the claim disputes autistic people’s autonomy, devalues their self-determination, and discredits their credibility (Yergeau, 2018). Consequently, numerous autistic authors have decried the claim, reporting that it “perpetuates stereotypes and oversimplifications [with] the potential for tremendous harm” (Cohen-Rottenberg, 2011); that it has already “harmed … countless autistic individuals” (VisualVox, 2017); and that “its continued perpetuation will continue to be damaging to autistic people” (Nicholson, 2013). We, therefore, call for considerably greater caution before endorsing the claim that autistic people lack a theory of mind.

## Figures and Tables

**Table 1:** Researchers’ Claims That Lack of Theory of Mind Is Specific to Autism

Citation	Quotation
Baron-Cohen (1988, p. 393)	“autistic children of normal intelligence failed to demonstrate that they could distinguish their own belief from someone else’s (Baron-Cohen, Leslie, & Frith, 1985, 1986). This is seen as **an autism-specific deficit**.”
Baron-Cohen (1989a, p. 188)	“What they seem to have **specific difficulty** with is understanding and predicting behavior in situations in which covert mental state attributions are required (Baron-Cohen, 1989a, 1989b, in press; Baron-Cohen et al., 1985, 1986; Leslie & Frith, 1988)”
Baron-Cohen (1989b, p. 294)	“The search for why a theory of mind fails to develop or is severely delayed in autism remains a key question for future research, and raises the clinical issue of whether any intervention could reduce **this specific delay**.”
Baron-Cohen (1989c, p. 200)	“the theory of mind hypothesis never set out to explain repetitive behaviours or phenomena other than the **autism-specific**, communicative and imaginative impairments”
Baron-Cohen (1990, pp. 81, 84)	“There is indeed an **autism-specific** cognitive deficit in this domain”; “The data from the control groups further reveals that such a deficit must be **autism-specific**, rather than the result of general developmental delay”
Baron-Cohen (1991a, p. 249)	“The general assumption in the specific developmental delay theory is that autistic children’s physical-causal knowledge is mental age appropriate and the only delayed aspect of their development that is **specific to autism** is in their theory of mind.”
Baron-Cohen (1991b, pp. 35, 47)	“the theory of mind deficit appears to be **highly specific**”; “they seem to have a **specific inability** to understand the ‘representational mind’”
Baron-Cohen (1991c, p. 312)	“the present results are therefore consistent with the hypothesis that the deficit in the development of a theory of mind in autism is **highly specific**”
Baron-Cohen (2000b, p. 15)	“children [with other developmental disabilities] may also have equivalent difficulty on ‘control’ tasks such as the False Photograph Task … whilst children with autism may show a **specific deficit** only on the theory of mind task”
Baron-Cohen (2001, p. 179)	“children [with other developmental disabilities] may also have equivalent difficulty on ‘control’ tasks such as the False Photograph Task … while children with autism may show a **specific deficit** only on the theory of mind task”
Baron-Cohen and Swettenham (1997, p. 883)	“We can therefore interpret these results in terms of there being **a specific developmental delay** in theory of mind at a number of different points.”
Baron-Cohen, Campbell, Karmiloff-Smith, Grant, and Walker (1995, p. 392)	“Results from both conditions thus provided converging evidence for an **autism specific deficit** in inferring when a person is thinking”
Baron-Cohen et al. (1985, pp. 37, 44)	“Thus the dysfunction we have postulated and demonstrated is … **specific to autism**”; “We conclude that the failure shown by the autistic children in our experiment constitutes a **specific deficit**”
Baron-Cohen, Leslie, and Frith (1986, p. 121)	“These results confirm and extend the findings of Baron-Cohen et al. (1985) that autistic children show a **specific deficit** in employing a ‘theory of mind’”
Baron-Cohen, Ring, Moriarty, Schmitz, Costa, and Ell (1994, p. 642)	“This deficit is further evidence for an **autism-specific** impairment in the child’s concept of mind”
Frith and Happé (1994, p. 126)	“At present, all the evidence suggests that we should retain the idea of a modular and **specific mentalizing deficit** in our causal explanation of the triad of impairments in autism.”
Leslie and Thaiss (1992, p. 226)	“We argue that children are equipped with a domain-specific processing mechanism (“ToMM”) which allows the child to attend to mental states, which … is **specifically impaired in autism**.”

*Note*. ToMM = theory of mind mechanism.

**Table 2:** Examples of Popular Theory-of-Mind Tasks

Type of task	Example
False Belief task (first-order)	Participant is shown a container with which they’d be familiar, for example, a closed bag of M&M candies. Participant is asked to predict what’s inside. The bag is opened, and the participant is shown that their belief about the contents was false: The bag doesn’t contain M&M candies; instead, it contains erasers. Participant is asked “What did you think would be inside the bag before I opened it?” If participant answers with the name of the bag’s actual content (e.g., erasers) rather than the name of the bag’s expected content (e.g., candy), the participant fails the false belief task.
False Belief task (second-order)	Similar to a first-order False Belief task (as illustrated above), except that the participant is asked, “What do you think another person would think would be inside the box before I opened it?”
Strange Stories task	Participant listens to a spoken story that contains a spoken deception (e.g., a lie, white lie, pretense, or double-bluff), a figure of speech (e.g., a metaphor or irony), a misunderstanding, persuasion, or the like. Participant is required to orally explain why the person said what they said and what they were thinking when they said it.
Faux Pas task	Participant listens to a spoken story that contains a social interaction, such as a person showing newly bought curtains to a friend, who says they don’t like the curtains. Participant is required to identify whether “someone said something that they shouldn’t have” and, if so, to orally explain why the person said something that they shouldn’t have, what they should have said instead, and what the person and their friend must have been thinking when the person said what they said.
Animated Triangles task	Participant views a series of animations with geometric triangles. After each animation, the participant is asked to orally explain “What happened in the animation?” Unknown to the participant, their oral answers are scored according to how likely they are to interpret the animated triangles as humans interacting and the number of emotional terms they provide in their oral explanation (e.g., if they say that one triangle was bullying another triangle).
Reading-the-Mind-in-the-Eye task	Participant views only the eye region of numerous black and white photographs and for each photograph is required to select one emotional expression from a set of four emotion terms (e.g., terrified, upset, annoyed, or arrogant).

**Table 3:** Researchers’ Claims That Lack of Theory of Mind Is Universal in Autism

Citation	Quotation
Baron-Cohen (1991b, pp. 47–48)	“the data reported here are consistent with the hypothesis that in **all cases of autism** there may be specific delay and deviance in the development of a theory of mind”
Baron-Cohen (2000b, p. 16)	“Mindreading deficits in autism spectrum conditions appear to be early occurring (from at least the end of the first year of life, if one includes joint attention deficits) and **universal** (if one tests for these either at the right point in development, or in the case of high-functioning, older subjects by using sensitive, age-appropriate tests).”
Baron-Cohen (2001a, pp. 169, 180)	“theory of mind difficulties seem to be **universal** among such individuals”
	“Mindreading deficits in autism-spectrum conditions appear to be early occurring (from at least the end of the first year of life, if one includes joint attention^4^ deficits) and **universal** (if one tests for these either at the right point in development or, in the case of high-functioning, older subjects, by using sensitive, age appropriate tests).”
Baron-Cohen (2001b, pp. 3, 14)	“theory of mind difficulties seem to be **universal** among such individuals” “Mindreading deficits in autism spectrum conditions appear to be early occurring (from at least the end of the first year of life, if one includes joint attention1 deficits) and **universal** (if one tests for these either at the right point in development, or in the case of high-functioning, older subjects by using sensitive, age-appropriate tests).
Baron-Cohen (2008a, p. 61)	“A strength of the mindblindness theory is that it can make sense of the social and communication difficulties in autism and Asperger syndrome, and that it is **universal in applying to all individuals on the autistic spectrum.”**
Baron-Cohen (2008b, p. 113)	“A strength of the mindblindness theory is that it can make sense of the social and communication difficulties in autism and Asperger’s syndrome, and that it is **universal in applying to all individuals on the autistic spectrum.”**
Baron-Cohen (2009, p. 70)	“degrees of mind-blindness are **universal in applying to all individuals on the autistic spectrum**, in that when age and mental-age-appropriate tests are used, deficits are found across the life span and independent of IQ”
Baron-Cohen (2010, p. 169)	“A strength of the mindblindness theory is that it can make sense of the social and communication difficulties in ASC [autism spectrum conditions], and that it is **universal in applying to all individuals on the autistic spectrum.”**
Baron-Cohen (2011a, p. 40)	“Two strengths of the mindblindness theory are that it can make sense of the social and communication difficulties in autism and Asperger’s syndrome and that it is **universal in applying to all individuals on the autistic spectrum.”**
Baron-Cohen (2011b, p. 629)	“Two strengths of the mindblindness theory are that it can make sense of the social and communication difficulties in autism and Asperger syndrome and that it is **universal in applying to all individuals on the autistic spectrum.”**
Baron-Cohen, Bolton, Wheelwright, Scahill, Short, Mead, and Smith (1998, p. 297)	“This impaired folk psychology appears to be **universal in autism,** even amongst adults with autism who have otherwise normal intelligence, though subtle tests of mind-reading are needed to reveal this … For this reason, autism has been characterized as involving degrees of ‘mindblindness’“
Becchio, Pierno, Mari, Lusher, and Castiello (2007, p. 2408)	“Autism has been **universally** and characteristically described as a dysfunction in ‘cognitive empathy’, i.e. the ability to represent the thoughts, desires and beliefs of others”
Happé (2001, p. 989)	“To date, a delay in theory of mind development appears to be **a universal feature of autism.”**

**Table 4:** Studies Demonstrating That Autistic People of All Ages Skillfully Understand Other Persons’ Intentions, Goals, and Desires

Study	Measure	Empirical finding
Aldridge, Stone, Sweeney, and Bower (2000)	Nonverbal behavior	Young, preverbal autistic children understand other people’s intentions “significantly better than the normally developing” children (p. 294).
Colombi et al. (2009)	Nonverbal behavior	Autistic preschool-age children understand other people’s intentions, a finding that “does not easily mesh with the line of reasoning” that claims autistic people have “deficits in the understanding of others’ mental states” (p. 157).
Carpenter, Pennington, and Rogers (2001)	Nonverbal behavior	Autistic pre-school-age children are not deficient “on any measure involving the understanding of others’ intentions” (p. 589).
Liebal, Colombi, Rogers, Warneken, and Tomasello (2008)	Nonverbal behavior	Autistic pre-school-age children “not only can understand another person’s goal,” but they are motivated to “help [that person] with that goal” (p. 229).
Falck-Ytter (2010)	Eye-tracking	Autistic pre-school-age children accurately “predict other people’s action goals” in ways that are “strikingly similar” to nonautistic preschoolers (p. 376).
Berger and Ingersoll (2014)	Nonverbal behavior	Autistic pre-school-age children “are able to use social-communicative cues [experimenter’s facial expressions] to understand intention” (p. 3204).
Fitzpatrick et al. (2013)	Nonverbal behavior	Autistic pre-school and early grade-school-age children “have the ability to understand intentions” and are “equivalent to typically developing children” on “social coordination tests” (pp. 1, 3, 9).
Kerr and Durkin (2004)	Spoken free response (drawings)	Autistic pre-school-age children understand “that (i) thought bubbles represent thought, (ii) thought bubbles can be used to infer an unknown reality, (iii) thoughts can be different, and (iv) thoughts can be false” (p. 646).
Li et al. (2019)	Eye-tracking and pupillometry	Autistic pre-school- and grade-school-age children are similar to typically developing children in their “unconscious sensitivity to agents’ intentions” (p. 9).
Green et al. (2017)	Multiple choice (photos)	Autistic grade-school-age children are as adept as nonautistic grade-school-age children at “identify[ing] … mutually voluntary interactions between intentional agents” (p. 406) and are characterized by a “similar … developmental trajectory” for this skill (p. 409).
Russell and Hill (2001)	Computer game, shooting game	Autistic grade-school-age children have “intact abilities in monitoring basic actions, intact abilities in reporting an intention, both for self and for another agent, and intact ability in reporting intended actions” (p. 317)
Vivanti et al. (2011)	Eye-tracking and nonverbal behavior	Autistic grade-school-age children “(a) consider situational constraints in order to understand the logic of an agent’s action and (b) show typical usage of the agent’s emotional expressions to infer his or her intentions” (p. 841).
McAleer, Kay, Pollick, and Rutherford (2011)	Multiple choice (videos)	Autistic adults demonstrate “no failure to recognize intent… . In no combination of variables did the autistic and nonautistic participants perform in a markedly different manner” (p. 1058).
Cole, Slocombe, and Barraclough (2018)	Multiple choice (videos)	Autistic adults do not differ from nonautistic adults in “implicit mentalizing” to make “social decisions [that] required the intentions of the actors to be inferred” (p. 3, 10).
Channon, Lagnado, Fitzpatrick, Drury, and Taylor (2011)	Multiple choice (written stories)	Autistic adults demonstrate “greater differentiation than controls between intentional and unintentional actions” and “between actions that the protagonists believed to be likely versus unlikely to lead to negative consequences” (p. 1534).
Sebanz, Knoblich, Stumpf, and Prinz (2005)	Response time	Autistic adults understand the intentions of a “co-actor … showing the same pattern of results as the matched control group” (p. 433).
Forgeot d’Arc et al. (2016)	Multiple choice (videos)	Autistic adults possess the same level of “spontaneous propensity to pursue goals that others pursue” as nonautistic adults possess (p. 1).
Hubert et al. (2007)	Spoken free response (videos)	Autistic adults perform equally “well in the description of basic actions” and “subjective states” as nonautistic adults, demonstrating that in autistic adults “intentionality is therefore well perceived” (p. 1390).
Ponnet, Buysse, Roeyers, and De Corte (2005)	Covertly videotaped interaction	Autistic adults do “not differ from the control adults in the ability to infer the thoughts and feelings of their interaction partner” (p. 595).

## Footnotes

1 We use identity-first language (e.g., autistic people, nonautistic people) rather than person-first language (e.g., people with autism, people without autism) because identity-first language is preferred by autistic people (Kenny et al., 2016), is recommended by APA (Dunn & Andrews, 2015), and is less likely to contribute to stigma (Gernsbacher, 2017).

2 All materials and data supporting the conclusions drawn in this article are available in Gernsbacher (2018a), which is a technical report available on the Open Science Framework.

3 For example, some researchers claim that autistic children “fail the false belief task because they lack the capacity to acquire a theory of mind,” whereas nonautistic children “fail the false belief task because of general task demands, because they don’t have a grasp of false belief, or both. But they surely have a ‘theory of mind’” (Bloom & German, 2000, p. B29).

4 Only sample sizes greater than 50 will be specified here; all other sample sizes are specified in Gernsbacher (2018a).

5 Baron-Cohen et al. (1997) agreed that the correlation between the Strange Stories task and the Reading-the-Mind-in-the-Eyes task “warrants direct testing” and promised that their article would provide that test (“to validate the Eyes Task as a theory of mind task, subjects in the two clinical groups were also tested on Happé’s [1994a] Strange Stories. In the case of the subjects with autism and Asperger Syndrome, this was part of a separate study [Jolliffe, 1997]”; pp. 815–816). Unfortunately, for neither the autistic nor the non-autistic participants is the correlation between Reading-the-Mind-in-the-Eyes and Strange Stories reported, in either Baron-Cohen et al.’s (1997) original article or Jolliffe’s (1997) “separate study.” Similarly, Baron-Cohen and colleagues (Vellante et al., 2013) claimed that “studies have found the [Reading-the-Mind-in-the-]Eyes test to be highly correlated with the Strange Stories test (Baron-Cohen, Wheelwright, Hill, et al., 2001)” (p. 329). Unfortunately, the article cited by Baron-Cohen and colleagues to support this claim (viz., Baron-Cohen et al., 2001) does not include the Strange Stories task (and Jolliffe & Baron-Cohen’s, 1999, article, which does include the Strange Stories task, does not include the Reading-the-Mind-in-the-Eyes task).

6 Only after later including “additional items specially designed to assess understanding of other minds in everyday life” could these researchers find any significant prediction of theory of mind for everyday social skills (Happé & Frith, 1996, p. 385).

## References

[R1] Abell F, & Hare DJ (2005). An experimental investigation of the phenomenology of delusional beliefs in people with Asperger syndrome. Autism, 9, 515–531. 10.1177/1362361305057857 16287703

[R2] Abu-Akel A (2003). The neurochemical hypothesis of “theory of mind.” Medical Hypotheses, 60, 382–386. 10.1016/S0306-9877(02)00406-1 12581615

[R3] Abu-Akel A, & Shamay-Tsoory S (2011). Neuroanatomical and neurochemical bases of theory of mind. Neuropsychologia, 49, 2971–2984. 10.1016/j.neuropsychologia.2011.07.012 21803062

[R4] Adler N, Nadler B, Eviatar Z, & Shamay-Tsoory SG (2010). The relationship between theory of mind and autobiographical memory in high-functioning autism and Asperger syndrome. Psychiatry Research, 178, 214–216. 10.1016/j.psychres.2009.11.015 20452047

[R5] Ahmed FS, & Miller LS (2011). Executive function mechanisms of theory of mind. Journal of Autism and Developmental Disorders, 41, 667–678. 10.1007/s10803-010-1087-7 20811770

[R6] Aldridge MA, Stone KR, Sweeney MH, & Bower TGR (2000). Preverbal children with autism understand the intentions of others. Developmental Science, 3, 294–301. 10.1111/1467-7687.00123

[R7] Ames DR, & Kammrath LK (2004). Mind-reading and metacognition: Narcissism, not actual competence, predicts self-estimated ability. Journal of Nonverbal Behavior, 28, 187–209. 10.1023/B:JONB.0000039649.20015.0e

[R8] Angus DJ, de Rosnay M, Lunenburg P, Meerum Terwogt M, & Begeer S (2015). Limitations in social anticipation are independent of imaginative and Theory of Mind abilities in children with autism but not in typically developing children. Autism, 19, 604–612. 10.1177/1362361314537911 24923896

[R9] Autism Society of Indiana. (n.d.) What is autism? 4 Common aspects of autism. Retrieved from http://www.autismsocietyofindiana.org/4-common-aspects-autism/

[R10] Autism Society of Minnesota. (2016). An explanation of affected characteristics. What is autism? Retrieved from https://www.ausm.org/about-ausm/what-is-autism.html

[R11] Autism Speaks. (2018, 8). Executive functioning and theory of mind. Retrieved from https://web.archive.org/web/20180826023601/https://www.autismspeaks.org/family-services/tool-kits/asperger-syndrome-and-high-functioning-autism-tool-kit/executive-functioni

[R12] Badenes LV, Clemente Estevan RA, & García Bacete FJ (2000). Theory of mind and peer rejection at school. Social Development, 9, 271–283. 10.1111/1467-9507.00125

[R13] Bailey A, Phillips W, & Rutter M (1996). Autism: Towards an integration of clinical, genetic, neuropsychological, and neurobiological perspectives. Journal of Child Psychology and Psychiatry, and Allied Disciplines, 37, 89–126. 10.1111/j.1469-7610.1996.tb01381.x 8655659

[R14] Barnbaum DR (2008). The ethics of autism: Among them, but not of them. Bloomington: Indiana University Press.

[R15] Barnes JL (2012). Fiction, imagination, and social cognition: Insights from autism. Poetics, 40, 299–316. 10.1016/j.poetic.2012.05.001

[R16] Baron-Cohen S (1988). Social and pragmatic deficits in autism: Cognitive or affective? Journal of Autism and Developmental Disorders, 18, 379–402. 10.1007/BF02212194 3049519

[R17] Baron-Cohen S (1989a). Joint-attention deficits in autism: Towards a cognitive analysis. Development and Psychopathology, 1, 185–189. 10.1017/S0954579400000377

[R18] Baron-Cohen S (1989b). The autistic child’s theory of mind: A case of specific developmental delay. Journal of Child Psychology and Psychiatry, and Allied Disciplines, 30, 285–297. 10.1111/j.1469-7610.1989.tb00241.x 2523408

[R19] Baron-Cohen S (1989c). The theory of mind hypothesis of autism: A reply to Boucher. International Journal of Language & Communication Disorders, 24, 199–200. 10.3109/13682828909011956 2605111

[R20] Baron-Cohen S (1990). Autism: A specific cognitive disorder of ‘mind-blindness’. International Review of Psychiatry, 2, 81–90. 10.3109/09540269009028274

[R21] Baron-Cohen S (1991a). Precursors to a theory of mind: Understanding attention in others In Whiten A (Ed.), Natural theories of mind: Evolution, development and simulation of everyday mindreading (pp. 233–251). Cambridge, MA: Basil Blackwell.

[R22] Baron-Cohen S (1991b). The development of a theory of mind in autism: Deviance and delay? The Psychiatric Clinics of North America, 14, 33–51. 10.1016/S0193-953X(18)30323-X 2047331

[R23] Baron-Cohen S (1991c). The theory of mind deficit in autism: How specific is it? British Journal of Developmental Psychology, 9, 301–314. 10.1111/j.2044-835X.1991.tb00879.x

[R24] Baron-Cohen S (1992). Out of sight or out of mind? Another look at deception in autism. Journal of Child Psychology and Psychiatry, and Allied Disciplines, 33, 1141–1155. 10.1111/j.1469-7610.1992.tb00934.x 1400697

[R25] Baron-Cohen S (1995). Mindblindness: An essay on autism and theory of mind. Cambridge, MA: Bradford.

[R26] Baron-Cohen S (2000a). The evolution of a theory of mind In Corballis M & Lea SEG (Eds.), The descent of mind: Psychological perspectives on hominid evolution (pp. 261–277). New York, NY: Oxford University Press 10.1093/acprof:oso/9780192632593.003.0013

[R27] Baron-Cohen S (2000b). Theory of mind and autism: A fifteen year review In Baron-Cohen S, Tager-Flusberg H & Cohen DJ (Eds.), Understanding other minds: Perspectives from developmental cognitive neuroscience (pp. 3–20). New York, NY: Oxford University Press.

[R28] Baron-Cohen S (2001a). Theory of mind and autism: A review. International Review of Research in Mental Retardation, 23, 169–184. 10.1016/S0074-7750(00)80010-5

[R29] Baron-Cohen S (2001b). Theory of mind in normal development and autism. Psychiatrie, Recherche et Intervention en Santé Mentale de l’Enfant, 34, 174–183.

[R30] Baron-Cohen S (2006). The hyper-systemizing, assortative mating theory of autism Progress in Neuro-Psychopharmacology & Biological Psychiatry, 30, 865–872. 10.1016/j.pnpbp.2006.01.010 16519981

[R31] Baron-Cohen S (2008a). Autism and Asperger syndrome. Oxford, United Kingdom: Oxford University Press.

[R32] Baron-Cohen S (2008b). Theories of the autistic mind. The Psychologist, 21, 112–116. Retrieved from https://thepsychologist.bps.org.uk/volume-21/edition-2/theories-autistic-mind

[R33] Baron-Cohen S (2009). Autism: The empathizing-systemizing (E-S) theory. Annals of the New York Academy of Sciences, 1156, 68–80. 10.1111/j.1749-6632.2009.04467.x 19338503

[R34] Baron-Cohen S (2010). Empathizing, systemizing, and the extreme male brain theory of autism. Progress in Brain Research, 186, 167–175. 10.1016/B978-0-444-53630-3.00011-7 21094892

[R35] Baron-Cohen S (2011a). The autistic mind: The empathizing-systematizing theory In Hollander E, Kolevzon A, & Coyle JT (Eds.), Textbook of autism spectrum disorders (pp. 39–48). Arlington, VA: American Psychiatric Publishing;

[R36] Baron-Cohen S (2011b). The empathizing-systemizing (E-S) theory of autism: A cognitive developmental account In Goswami U (Ed.), The Wiley-Blackwell handbook of childhood cognitive development (2nd ed., pp. 626–639). Hoboken, NJ: Wiley-Blackwell.

[R37] Baron-Cohen S, Bolton P, Wheelwright S, Scahill V, Short L, Mead G, & Smith A (1998). Does autism occur more often in families of physicists, engineers, and mathematicians? Autism, 2, 296–301. 10.1177/1362361398023008

[R38] Baron-Cohen S, Bowen DC, Holt RJ, Allison C, Auyeung B, Lombardo MV, … Lai MC (2015). The “Reading the Mind in the Eyes” Test: Complete absence of typical sex difference in ~400 men and women with autism. PLoS ONE, 10, e0136521 10.1371/journal.pone.0136521 26313946 PMC4552377

[R39] Baron-Cohen S, Campbell R, Karmiloff-Smith A, Grant J, & Walker J (1995). Are children with autism blind to the mentalistic significance of the eyes? British Journal of Developmental Psychology, 13, 379–398. 10.1111/j.2044-835X.1995.tb00687.x

[R40] Baron-Cohen S, Jolliffe T, Mortimore C,& Robertson M (1997). Another advanced test of theory of mind: Evidence from very high functioning adults with autism or Asperger syndrome. Journal of Child Psychology and Psychiatry, and Allied Disciplines, 38, 813–822. 10.1111/j.1469-7610.1997.tb01599.x 9363580

[R41] Baron-Cohen S, Leslie AM, & Frith U (1985). Does the autistic child have a “theory of mind”? Cognition, 21, 37–46. 10.1016/0010-0277(85)90022-8 2934210

[R42] Baron-Cohen S, Leslie AM, & Frith U (1986). Mechanical, behavioural and Intentional understanding of picture stories in autistic children. British Journal of Developmental Psychology, 4, 113–125. 10.1111/j.2044-835X.1986.tb01003.x

[R43] Baron-Cohen S, Ring H, Moriarty J, Schmitz B, Costa D, & Ell P (1994). Recognition of mental terms: Clinical findings in children with autism and a functional neuroimaging study of normal adults. British Journal of Psychiatry, 165, 640–649. 10.1192/bjp.165.5.640 7866679

[R44] Baron-Cohen S, & Swettenham J (1997). Theory of mind in autism: Its relationship to executive function and central coherence In Cohen DJ & Volkmar FR (Eds.), Handbook of autism and pervasive developmental disorders (2nd ed., pp. 880–893). New York, NY: Wiley.

[R45] Baron-Cohen S, Wheelwright S, Hill J, Raste Y, & Plumb I (2001). The “Reading the Mind in the Eyes” Test revised version: A study with normal adults, and adults with Asperger syndrome or high-functioning autism. Journal of Child Psychology and Psychiatry, and Allied Disciplines, 42, 241–251. 10.1111/1469-7610.00715 11280420

[R46] Baron-Cohen S, Wheelwright S, & Jolliffe T (1997). Is there a “language of the eyes”? Evidence from normal adults, and adults with autism or Asperger syndrome. Visual Cognition, 4, 311–331. 10.1080/713756761

[R47] Bauminger N, & Kasari C (1999). Brief report: Theory of mind in high-functioning children with autism. Journal of Autism and Developmental Disorders, 29, 81–86. 10.1023/A:1025974701090 10097997

[R48] Becchio C, Pierno A, Mari M, Lusher D, & Castiello U (2007). Motor contagion from gaze: The case of autism. Brain: A Journal of Neurology, 130, 2401–2411. 10.1093/brain/awm171 17711981

[R49] Begeer S, Malle BF, Nieuwland MS, & Keysar B (2010). Using theory of mind to represent and take part in social interactions: Comparing individuals with high-functioning autism and typically developing controls. European Journal of Developmental Psychology, 7, 104–122. 10.1080/17405620903024263

[R50] Bennett TA, Szatmari P, Bryson S, Duku E, Vaccarella L, & Tuff L (2013). Theory of mind, language and adaptive functioning in ASD: A neuroconstructivist perspective. Journal of the Canadian Academy of Child and Adolescent Psychiatry, 22, 13–19. Retrieved from https://www.ncbi.nlm.nih.gov/pmc/articles/PMC3565710/23390428 PMC3565710

[R51] Berger NI, & Ingersoll B (2014). A further investigation of goal-directed intention understanding in young children with autism spectrum disorders. Journal of Autism and Developmental Disorders, 44, 3204–3214. 10.1007/s10803-014-2181-z 25001543

[R52] Beversdorf DQ, Anderson JM, Manning SE, Anderson SL, Nordgren RE, Felopulos GJ, … Bauman ML (1998). The effect of semantic and emotional context on written recall for verbal language in high functioning adults with autism spectrum disorder. Journal of Neurology, Neurosurgery, and Psychiatry, 65, 685–692. 10.1136/jnnp.65.5.685 PMC2170365 9810938

[R53] Bloom P, & German TP (2000). Two reasons to abandon the false belief task as a test of theory of mind. Cognition, 77, B25–B31. 10.1016/S0010-0277(00)00096-2 10980256

[R54] Bora E, Vahip S, Gonul AS, Akdeniz F, Alkan M, Ogut M, & Eryavuz A (2005). Evidence for theory of mind deficits in euthymic patients with bipolar disorder. Acta Psychiatrica Scandinavica, 112, 110–116. 10.1111/j.1600-0447.2005.00570.x 15992392

[R55] Bosacki S, & Astington JW (1999). Theory of mind in preadolescence: Relations between social understanding and social competence. Social Development, 8, 237–255. 10.1111/1467-9507.00093

[R56] Botting N, & Conti-Ramsden G (2008). The role of language, social cognition, and social skill in the functional social outcomes of young adolescents with and without a history of SLI. British Journal of Developmental Psychology, 26, 281–300. 10.1348/026151007X235891

[R57] Boucher J (2012). Putting theory of mind in its place: Psychological explanations of the socio-emotional-communicative impairments in autistic spectrum disorder. Autism, 16, 226–246. 10.1177/1362361311430403 22297199

[R58] Bowler DM (1992). “Theory of mind” in Asperger’s syndrome. Journal of Child Psychology and Psychiatry, and Allied Disciplines, 33, 877–893. 10.1111/j.1469-7610.1992.tb01962.x 1378848

[R59] Boyer P (2000). Functional origins of religious concepts: Ontological and strategic selection in evolved minds. Journal of the Royal Anthropological Institute, 6, 195–214. 10.1111/1467-9655.00012

[R60] Brambring M, & Asbrock D (2010). Validity of false belief tasks in blind children. Journal of Autism and Developmental Disorders, 40, 1471–1484. 10.1007/s10803-010-1002-2 20379770

[R61] Brent E, Rios P, Happé F, & Charman T (2004). Performance of children with autism spectrum disorder on advanced theory of mind tasks. Autism, 8, 283–299. 10.1177/1362361304045217 15358871

[R62] Brewer N, Young RL, & Barnett E (2017). Measuring theory of mind in adults with autism spectrum disorder. Journal of Autism and Developmental Disorders, 47, 1927–1941. 10.1007/s10803-017-3080-x 28275927 PMC5487761

[R63] Brooks R, & Meltzoff AN (2015). Connecting the dots from infancy to childhood: A longitudinal study connecting gaze following, language, and explicit theory of mind. Journal of Experimental Child Psychology, 130, 67–78. 10.1016/j.jecp.2014.09.010 25462032 PMC7089676

[R64] Bryant L, Coffey A, Povinelli DJ, & Pruett JR Jr.. (2013). Theory of Mind experience sampling in typical adults. Consciousness and Cognition, 22, 697–707. 10.1016/j.concog.2013.04.005 23685620 PMC3778034

[R65] Buitelaar JK, van der Wees M, Swaab-Barneveld H, & van der Gaag RJ (1999a). Theory of mind and emotion-recognition functioning in autistic spectrum disorders and in psychiatric control and normal children. Development and Psychopathology, 11, 39–58. 10.1017/S0954579499001947 10208355

[R66] Buitelaar JK, van der Wees M, Swaab-Barneveld H, & van der Gaag RJ (1999b). Verbal memory and Performance IQ predict theory of mind and emotion recognition ability in children with autistic spectrum disorders and in psychiatric control children. Journal of Child Psychology and Psychiatry, and Allied Disciplines, 40, 869–881. 10.1111/1469-7610.00505 10509882

[R67] Burnside K, Wright K, & Poulin-Dubois D (2017). Social motivation and implicit theory of mind in children with autism spectrum disorder. Autism Research, 10, 1834–1844. 10.1002/aur.1836 28762662 PMC5772680

[R68] Caillies S, Hody A, & Calmus A (2012). Theory of mind and irony comprehension in children with cerebral palsy. Research in Developmental Disabilities, 33, 1380–1388. 10.1016/j.ridd.2012.03.012 22522196

[R69] Campbell LE, Stevens AF, McCabe K, Cruickshank L, Morris RG, Murphy DG, & Murphy KC (2011). Is theory of mind related to social dysfunction and emotional problems in 22q11.2 deletion syndrome (velo-cardio-facial syndrome)? Journal of Neurodevelopmental Disorders, 3, 152–161. 10.1007/s11689-011-9082-7 21544568 PMC3188292

[R70] Capage L, & Watson AC (2001). Individual differences in theory of mind, aggressive behavior, and social skills in young children. Early Education and Development, 12, 613–628. 10.1207/s15566935eed1204_7

[R71] Carpenter M, Pennington BF, & Rogers SJ (2001). Understanding of others’ intentions in children with autism. Journal of Autism and Developmental Disorders, 31, 589–599. 10.1023/A:1013251112392 11814270

[R72] Carroll JM, & Chiew KY (2006). Sex and discipline differences in empathising, systemising and autistic symptomatology: Evidence from a student population. Journal of Autism and Developmental Disorders, 36, 949–957. 10.1007/s10803-006-0127-9 16897399

[R73] Carter v. Superintendent, No. 3:09-CV-393-TS (N. D. Ind. 2011)

[R74] Castelli I, Pini A, Alberoni M, Liverta-Sempio O, Baglio F, Massaro D, … Nemni R (2011). Mapping levels of theory of mind in Alzheimer’s disease: A preliminary study. Aging & Mental Health, 15, 157–168. 10.1080/13607863.2010.513038 21140304

[R75] Channon S, Lagnado D, Fitzpatrick S, Drury H, & Taylor I (2011). Judgments of cause and blame: Sensitivity to intentionality in Asperger’s syndrome. Journal of Autism and Developmental Disorders, 41, 1534–1542. 10.1007/s10803-011-1180-6 21287254

[R76] Charman T (2000). Theory of mind and the early diagnosis of autism In Baron-Cohen S, Tager-Flusberg H, & Cohen DJ (Eds.), Understanding other minds: Perspectives from developmental cognitive neuroscience (pp. 422–441). New York, NY: Oxford University Press.

[R77] Charman T, & Campbell A (1997). Reliability of theory of mind task performance by individuals with a learning disability: A research note. Journal of Child Psychology and Psychiatry, and Allied Disciplines, 38, 725–730. 10.1111/j.1469-7610.1997.tb01699.x 9315982

[R78] Chen K-W, Lee S-C, Chiang H-Y, Syu Y-C, Yu X-X, & Hsieh C-L (2017). Psychometric properties of three measures assessing advanced theory of mind: Evidence from people with schizophrenia. Psychiatry Research, 257, 490–496. 10.1016/j.psychres.2017.08.026 28841511

[R79] Chin HY, & Bernard-Opitz V (2000). Teaching conversational skills to children with autism: Effect on the development of a theory of mind. Journal of Autism and Developmental Disorders, 30, 569–583. 10.1023/A:1005639427185 11261468

[R80] Clegg J, Hollis C, Mawhood L, & Rutter M (2005). Developmental language disorders: A follow-up in later adult life: Cognitive, language and psychosocial outcomes. Journal of Child Psychology and Psychiatry, and Allied Disciplines, 46, 128–149. 10.1111/j.1469-7610.2004.00342.x 15679523

[R81] Clemmensen L, Bartels-Velthuis AA, Jespersen RA, van Os J, Blijd-Hoogewys EMA, Ankerstrøm L, … Jepsen JRM (2016). A psychometric evaluation of the Danish version of the theory of mind storybook for 8–14-year-old children. Frontiers in Psychology, 7, 330 10.3389/fpsyg.2016.00330 27014139 PMC4781859

[R82] Cohen-Rottenberg R (2011). Unwarranted conclusions and the potential for harm: My reply to Simon Baron-Cohen [Blog post]. Retrieved from http://autismandempathyblog.wordpress.com/unwarranted-conclusions-and-the-potential-for-harm-my-reply-to-simon-baron-cohen/

[R83] Cole EJ, Slocombe KE, & Barraclough NE (2018). Abilities to explicitly and implicitly infer intentions from actions in adults with autism spectrum disorder. Journal of Autism and Developmental Disorders, 48, 1712–1726. 10.1007/s10803-017-3425-5 29214604 PMC5889782

[R84] Colombi C, Liebal K, Tomasello M, Young G, Warneken F, & Rogers SJ (2009). Examining correlates of cooperation in autism: Imitation, joint attention, and understanding intentions. Autism, 13, 143–163. 19261685 10.1177/1362361308098514

[R85] Coon D, Mitterer JO, & Martini TS (2018). Psychology: Modules for active learning (14th ed.). Independence, KY: Cengage Learning.

[R86] Cornish K, Burack JA, Rahman A, Munir F, Russo N, & Grant C (2005). Theory of mind deficits in children with fragile X syndrome. Journal of Intellectual Disability Research, 49, 372–378. 10.1111/j.1365-2788.2005.00678.x 15817054

[R87] Dahlgren S, Dahlgren Sandberg A, & Hjelmquist E (2003). The non-specificity of theory of mind deficits: Evidence from children with communicative disabilities. The European Journal of Cognitive Psychology, 15, 129–155. 10.1080/09541440303601

[R88] Dahlgren SO, & Trillingsgaard A (1996). Theory of mind in non-retarded children with autism and Asperger’s syndrome: A research note. Journal of Child Psychology and Psychiatry, and Allied Disciplines, 37, 759–763. 10.1111/j.1469-7610.1996.tb01469.x 8894958

[R89] de Lima Velloso R, Duarte CP, & Schwartzman JS (2013). Evaluation of the theory of mind in autism spectrum disorders with the Strange Stories test. Arquivos de Neuro-Psiquiatria, 71, 871–876. 10.1590/0004-282X20130171 24394874

[R90] Devine RT, & Hughes C (2013). Silent films and strange stories: Theory of mind, gender, and social experiences in middle childhood. Child Development, 84, 989–1003. 10.1111/cdev.12017 23199139

[R91] Ding XP, Wellman HM, Wang Y, Fu G, & Lee K (2015). Theory-of-mind training causes honest young children to lie. Psychological Science, 26, 1812–1821. 10.1177/0956797615604628 26431737 PMC4636928

[R92] Dinishak J, & Akhtar N (2013). A critical examination of mindblindness as a metaphor for autism. Child Development Perspectives, 7, 110–114. 10.1111/cdep.12026

[R93] Dorris L, Espie CAE, Knott F, & Salt J (2004). Mind-reading difficulties in the siblings of people with Asperger’s syndrome: Evidence for a genetic influence in the abnormal development of a specific cognitive domain. Journal of Child Psychology and Psychiatry, and Allied Disciplines, 45, 412–418. 10.1111/j.1469-7610.2004.00232.x 14982254

[R94] Dunn DS, & Andrews EE (2015). Person-first and identity-first language: Developing psychologists’ cultural competence using disability language. American Psychologist, 70, 255–264. 10.1037/a0038636 25642702

[R95] Duval C, Piolino P, Bejanin A, Eustache F, & Desgranges B (2011). Age effects on different components of theory of mind. Consciousness and Cognition, 20, 627–642. 10.1016/j.concog.2010.10.025 21111637

[R96] Dyck MJ, Ferguson K, & Shochet IM (2001). Do autism spectrum disorders differ from each other and from non-spectrum disorders on emotion recognition tests? European Child & Adolescent Psychiatry, 10, 105–116. 10.1007/s007870170033 11469282

[R97] Dziobek I, Fleck S, Kalbe E, Rogers K, Hassenstab J, Brand M, … Convit A (2006). Introducing MASC: A movie for the assessment of social cognition. Journal of Autism and Developmental Disorders, 36, 623–636. 10.1007/s10803-006-0107-0 16755332

[R98] Eisenmajer R, & Prior M (1991). Cognitive linguistic correlates of “theory of mind” ability in autistic children. British Journal of Developmental Psychology, 9, 351–364. 10.1111/j.2044-835X.1991.tb00882.x

[R99] Falck-Ytter T (2010). Young children with autism spectrum disorder use predictive eye movements in action observation. Biology Letters, 6, 375–378. 10.1098/rsbl.2009.0897 20031980 PMC2880053

[R100] Farrant A, Morris RG, Russell T, Elwes R, Akanuma N, Alarcon G, & Koutroumanidis M (2005). Social cognition in frontal lobe epilepsy. Epilepsy & Behavior, 7, 506–516. 10.1016/j.yebeh.2005.07.018 16165399

[R101] Ferguson FJ, & Austin EJ (2010). Associations of trait and ability emotional intelligence with performance on Theory of Mind tasks in an adult sample. Personality and Individual Differences, 49, 414–418. 10.1016/j.paid.2010.04.009

[R102] Figueras-Costa B, & Harris P (2001). Theory of mind development in deaf children: A nonverbal test of false-belief understanding. Journal of Deaf Studies and Deaf Education, 6, 92–102. 10.1093/deafed/6.2.92 15451854

[R103] Fitzpatrick P, Diorio R, Richardson MJ, & Schmidt RC (2013). Dynamical methods for evaluating the time-dependent unfolding of social coordination in children with autism. Frontiers in Integrative Neuroscience, 7, 21 10.3389/fnint.2013.00021 23580133 PMC3619188

[R104] Fombonne E, Siddons F, Achard S, Frith U, & Happé F (1994). Adaptive behaviour and theory of mind in autism. European Child & Adolescent Psychiatry, 3, 176–186. 10.1007/BF02720324 29871424

[R105] Forgeot d’ Arc B, Vinckier F, Lebreton M, Soulières MI, Mottron L, & Pessiglione M (2016). Mimetic desire in autism spectrum disorder. Molecular Autism, 7, 45 10.1186/s13229-016-0107-7 27826407 PMC5100325

[R106] Franco A, Malhotra N, & Simonovits G (2014). Social science. Publication bias in the social sciences: Unlocking the file drawer. Science, 345, 1502–1505. 10.1126/science.1255484 25170047

[R107] Frith U, & Happé F (1994). Autism: Beyond “theory of mind.” Cognition, 50, 115–132. 10.1016/0010-0277(94)90024-8 8039356

[R108] Frith U, Happé F, & Siddons F (1994). Autism and theory of mind in everyday life. Social Development, 3, 108–124. 10.1111/j.1467-9507.1994.tb00031.x

[R109] Frölander HE, Möller C, Marshall JD, Sundqvist A, Rönnåsen B, Falkensson L, & Lyxell B (2014). Theory-of-mind in adolescents and young adults with Alstrom syndrome. International Journal of Pediatric Otorhinolaryngology, 78, 530–536. 10.1016/j.ijporl.2013.12.038 24485176

[R110] Frye D, Zelazo PD, & Burack JA (1998). Cognitive complexity and control: I. Theory of mind in typical and atypical development. Current Directions in Psychological Science, 7, 116–121. 10.1111/1467-8721.ep10774754

[R111] Gernsbacher MA (2006). Toward a behavior of reciprocity. The Journal of Developmental Processes, 1, 139–152. Retrieved from https://www.ncbi.nlm.nih.gov/pmc/articles/PMC4296736 25598865 PMC4296736

[R112] Gernsbacher MA (2007). On not being human. APS Observer, 20, 5–32. Retrieved from https://www.ncbi.nlm.nih.gov/pmc/articles/PMC4266404/PMC4266404 25520547

[R113] Gernsbacher MA (2017). Editorial Perspective: The use of person-first language in scholarly writing may accentuate stigma. Journal of Child Psychology and Psychiatry, and Allied Disciplines, 58, 859–861. 10.1111/jcpp.12706 PMC5545113 28621486

[R114] Gernsbacher MA (2018a). Critical review of autism and theory and mind: A technical report. Open Science Framework. 10.17605/OSF.IO/3R2QY

[R115] Gernsbacher MA (2018b). Rewarding research transparency. Trends in Cognitive Sciences, 22, 953–956. 10.1016/j.tics.2018.07.002 30041865 PMC6195839

[R116] Gernsbacher MA (2018c). Three ways to make replication mainstream. Behavioral and Brain Sciences, 41, e129 10.1017/S0140525X1800064X 30757985 PMC6375101

[R117] Gernsbacher MA (2018d). Writing empirical articles: Transparency, reproducibility, clarity, and memorability. Advances in Methods and Practices in Psychological Science, 1, 403–414. 10.1177/2515245918754485 30775689 PMC6377209

[R118] Gernsbacher MA, & Frymiare JL (2005). Does the autistic brain lack core modules? The Journal of Developmental and Learning Disorders, 9, 3–16. Retrieved from https://www.ncbi.nlm.nih.gov/pmc/articles/PMC4266369/25520587 PMC4266369

[R119] Gernsbacher MA, Geye HM, & Ellis Weismer S (2005). The role of language and communication impairments within autism In Fletcher P & Miller JF (Eds.), Language disorders and developmental theory (pp. 73–93). Philadelphia, PA: John Benjamins 10.1075/tilar.4.06ger

[R120] Gernsbacher MA, Morson EM, & Grace EJ (2016). Language and speech in autism. Annual Review of Linguistics, 2, 413–425. 10.1146/annurev-linguistics-030514-124824 PMC5260808 28127576

[R121] Gernsbacher MA, & Pripas-Kapit SR (2012). Who’s missing the point? A commentary on claims that autistic persons have a specific deficit in figurative language comprehension. Metaphor and Symbol, 27, 93–105. 10.1080/10926488.2012.656255 25339845 PMC4203428

[R122] Gernsbacher MA, Stevenson JL, & Dern S (2017). Specificity, contexts, and reference groups matter when assessing autistic traits. PLoS ONE, 12, e0171931 10.1371/journal.pone.0171931 28192464 PMC5305234

[R123] Gerrans P (2002). The theory of mind module in evolutionary psychology. Biology & Philosophy, 17, 305–321. 10.1023/A:1020183525825

[R124] Gillott A, Furniss F, & Walter A (2004). Theory of mind ability in children with specific language impairment. Child Language Teaching and Therapy, 20, 1–11. 10.1191/0265659004ct260oa

[R125] Gökçen E, Frederickson N, & Petrides KV (2016). Theory of mind and executive control deficits in typically developing adults and adolescents with high levels of autism traits. Journal of Autism and Developmental Disorders, 46, 2072–2087. 10.1007/s10803-016-2735-3 26886468 PMC4860196

[R126] Gooding DC, & Pflum MJ (2011). Theory of mind and psychometric schizotypy. Psychiatry Research, 188, 217–223. 10.1016/j.psychres.2011.04.029 21596443

[R127] Goodman L (2010). Rebellious identification, or, How I learned to stop worrying and love Arabella. Narrative, 18, 163–178. 10.1353/nar.0.0044

[R128] Green AE, Kenworthy L, Gallagher NM, Antezana L, Mosner MG, Krieg S, … Yerys BE (2017). Social analogical reasoning in school-aged children with autism spectrum disorder and typically developing peers. Autism, 21, 403–411. 10.1177/1362361316644728 27178998 PMC6085745

[R129] Green S, Pring L, & Swettenham J (2004). An investigation of first-order False Belief understanding of children with congenital profound visual impairment. British Journal of Developmental Psychology, 22, 1–17. 10.1348/026151004772901087

[R130] Happé FGE (1994a). An advanced test of theory of mind: Understanding of story characters’ thoughts and feelings by able autistic, mentally handicapped, and normal children and adults. Journal of Autism and Developmental Disorders, 24, 129–154. 10.1007/BF02172093 8040158

[R131] Happé FGE (1994b). Annotation: Current psychological theories of autism: The “theory of mind” account and rival theories. Journal of Child Psychology and Psychiatry, and Allied Disciplines, 35, 215–229. 10.1111/j.1469-7610.1994.tb01159.x 8188796

[R132] Happé FGE (1995). The role of age and verbal ability in the theory of mind task performance of subjects with autism. Child Development, 66, 843–855. 10.2307/1131954 7789204

[R133] Happé F (2001). Autistic disorder: Psychological In Smelser NJ & Baltes PB (Eds.), International encyclopedia of the social & behavioral sciences (2nd ed., pp. 987–991). Oxford, United Kingdom: Elsevier 10.1016/B0-08-043076-7/01347-4

[R134] Happé F, & Frith U (1996). Theory of mind and social impairment in children with conduct disorder. British Journal of Developmental Psychology, 14, 385–398. 10.1111/j.2044-835X.1996.tb00713.x

[R135] Higher Education Research Institute. (2017). College senior survey. Los Angeles, CA: University of California, Los Angeles Graduate School of Education and Information Studies.

[R136] Hollocks MJ, Jones CR, Pickles A, Baird G, Happé F, Charman T, & Simonoff E (2014). The association between social cognition and executive functioning and symptoms of anxiety and depression in adolescents with autism spectrum disorders. Autism Research, 7, 216–228. 10.1002/aur.1361 24737743

[R137] Hubert B, Wicker B, Moore DG, Monfardini E, Duverger H, Da Fonseca D, & Deruelle C (2007). Recognition of emotional and non-emotional biological motion in individuals with autistic spectrum disorders. Journal of Autism and Developmental Disorders, 37, 1386–1392. 10.1007/s10803-006-0275-y 17160459

[R138] Hughes C (1998). Executive function in preschoolers: Links with theory of mind and verbal ability. British Journal of Developmental Psychology, 16, 233–253. 10.1111/j.2044-835X.1998.tb00921.x

[R139] Hughes C, & Ensor R (2005). Executive function and theory of mind in 2 year olds: A family affair? Developmental Neuropsychology, 28, 645–668. 10.1207/s15326942dn2802_5 16144431

[R140] Hughes C, Soares-Boucaud I, Hochmann J, & Frith U (1997). Social behaviour in pervasive developmental disorders: Effects of informant, group and “theory-of-mind.” European Child & Adolescent Psychiatry, 6, 191–198. 9442997 10.1007/BF00539925

[R141] Hünefeldt T, Laghi F, Ortu F, & Belardinelli MO (2013). The relationship between “theory of mind” and attachment-related anxiety and avoidance in Italian adolescents. Journal of Adolescence, 36, 613–621. 10.1016/j.adolescence.2013.03.012 23595130

[R142] Hutchins TL, Prelock PA, & Bonazinga L (2012). Psychometric evaluation of the Theory of Mind Inventory (ToMI): A study of typically developing children and children with autism spectrum disorder. Journal of Autism and Developmental Disorders, 42, 327–341. 10.1007/s10803-011-1244-7 21484516

[R143] Ioannidis JPA (2008). Finding large effect sizes—Good news or bad news? The Psychologist, 21, 690–691.

[R144] Jackson AL (2001). Language facility and theory of mind development in deaf children. Journal of Deaf Studies and Deaf Education, 6, 161–176. 10.1093/deafed/6.3.161 15451847

[R145] Jenkins JM, & Astington JW (1996). Cognitive factors and family structure associated with theory of mind development in young children. Developmental Psychology, 32, 70–78. 10.1037/0012-1649.32.1.70

[R146] Jolliffe T (1997). Central coherence dysfunction in autistic spectrum disorder (Doctoral dissertation) Cambridge University, Cambridge, England.

[R147] Jolliffe T, & Baron-Cohen S (1999). The Strange Stories Test: A replication with high-functioning adults with autism or Asperger syndrome. Journal of Autism and Developmental Disorders, 29, 395–406. 10.1023/A:1023082928366 10587886

[R148] Jones CRG, Simonoff E, Baird G, Pickles A, Marsden AJS, Tregay J, & Charman T (2018). The association between theory of mind, executive function, and the symptoms of autism spectrum disorder. Autism Research, 11, 95–109. 10.1002/aur.1873 28945319

[R149] Joseph RM, & Tager-Flusberg H (2004). The relationship of theory of mind and executive functions to symptom type and severity in children with autism. Development and Psychopathology, 16, 137–155. 10.1017/S095457940404444X 15115068 PMC1201455

[R150] Kaland N, Callesen K, Møller-Nielsen A, Mortensen EL, & Smith L (2008). Performance of children and adolescents with Asperger syndrome or high-functioning autism on advanced theory of mind tasks. Journal of Autism and Developmental Disorders, 38, 1112–1123. 10.1007/s10803-007-0496-8 18058213

[R151] Kaland N, Møller-Nielsen A, Smith L, Mortensen EL, Callesen K, & Gottlieb D (2005). The Strange Stories test: A replication study of children and adolescents with Asperger syndrome. European Journal of Child and Adolescent Psychiatry, 14, 73–82. 10.1007/s00787-005-0434-2 15793686

[R152] Kellogg RT (2007). Fundamentals of cognitive psychology. Thousand Oaks, CA: Sage.

[R153] Kenny L, Hattersley C, Molins B, Buckley C, Povey C, & Pellicano E (2016). Which terms should be used to describe autism? Perspectives from the U.K. autism community. Autism, 20, 442–462. 10.1177/1362361315588200 26134030

[R154] Kerr S, & Durkin K (2004). Understanding of thought bubbles as mental representations in children with autism: Implications for theory of mind. Journal of Autism and Developmental Disorders, 34, 637–648. 10.1007/s10803-004-5285-z 15679184

[R155] Kirk S, Gallagher J, Coleman MR, & Anastasiow NJ (2008). Educating exceptional children. Independence, KY: Cengage Learning.

[R156] Kristen S, Rossmann F, & Sodian B (2014). Theory of own mind and autobiographical memory in adults with ASD. Research in Autism Spectrum Disorders, 8, 827–837. 10.1016/j.rasd.2014.03.009

[R157] Kristen S, Vuori M, & Sodian B (2015). “I love the cute caterpillar!” Autistic children’s production of internal state language across contexts and relations to joint attention and theory of mind. Research in Autism Spectrum Disorders, 12, 22–33. 10.1016/j.rasd.2014.12.006

[R158] Kulke L, von Duhn B, Schneider D, & Rakoczy H (2018). Is implicit theory of mind a real and robust phenomenon? Results from a systematic replication study. Psychological Science, 29, 888–900. 10.1177/0956797617747090 29659340

[R159] Kunihira Y, Senju A, Dairoku H, Wakabayashi A, & Hasegawa T (2006). “Autistic” traits in non-autistic Japanese populations: Relationships with personality traits and cognitive ability. Journal of Autism and Developmental Disorders, 36, 553–566. 10.1007/s10803-006-0094-1 16602034

[R160] Lalonde CE, & Chandler MJ (1995). False belief understanding goes to school: On the social-emotional consequences of coming early or late to a first theory of mind. Cognition and Emotion, 9, 167–185. 10.1080/02699939508409007

[R161] Lawrence EJ, Shaw P, Baker D, Baron-Cohen S, & David AS (2004). Measuring empathy: Reliability and validity of the Empathy Quotient. Psychological Medicine, 34, 911–919. 10.1017/S0033291703001624 15500311

[R162] Leekam SR, & Prior M (1994). Can autistic children distinguish lies from jokes? A second look at second-order belief attribution. Journal of Child Psychology and Psychiatry, and Allied Disciplines, 35, 901–915. 10.1111/j.1469-7610.1994.tb02301.x 7962247

[R163] Leslie AM, & Frith U (1988). Autistic children’s understanding of seeing, knowing, and believing. British Journal of Developmental Psychology, 6, 315–324. 10.1111/j.2044-835X.1988.tb01104.x

[R164] Leslie AM, & Thaiss L (1992). Domain specificity in conceptual development: Neuropsychological evidence from autism. Cognition, 43, 225–251. 10.1016/0010-0277(92)90013-8 1643814

[R165] Lew AR, Lewis C, Lunn J, Tomlin P, Basu H, Roach J, … Martland T (2015). Social cognition in children with epilepsy in mainstream education. Developmental Medicine and Child Neurology, 57, 53–59. 10.1111/dmcn.12613 25330820 PMC4328452

[R166] Lewis C, Freeman NH, Kyriakidou C, Maridaki-Kassotaki K, & Berridge DM (1996). Social influences on false belief access: Specific sibling influences or general apprenticeship? Child Development, 67, 2930–2947. 10.2307/1131760 9071766

[R167] Li T, Decety J, Hu X, Li J, Lin J, & Yi L (2019). Third-party sociomoral evaluations in children with autism spectrum disorder. Child Development. Advance online publication. 10.1111/cdev.13206 30620404

[R168] Li X, Wang K, Wang F, Tao Q, Xie Y, & Cheng Q (2013). Aging of theory of mind: The influence of educational level and cognitive processing. International Journal of Psychology, 48, 715–727. 10.1080/00207594.2012.673724 22515730

[R169] Liebal K, Colombi C, Rogers SJ, Warneken F, & Tomasello M (2008). Helping and cooperation in children with autism. Journal of Autism and Developmental Disorders, 38, 224–238. 10.1007/s10803-007-0381-5 17694374 PMC2758368

[R170] Lo ST, Siemensma E, Collin P, & Hokken-Koelega A (2013). Impaired theory of mind and symptoms of Autism Spectrum Disorder in children with Prader-Willi syndrome. Research in Developmental Disabilities, 34, 2764–2773. 10.1016/j.ridd.2013.05.024 23792373

[R171] Loftis SF (2015). Imagining autism: Fiction and stereotypes on the spectrum. Bloomington, IN: Indiana University Press.

[R172] Lombardo MV, Lai MC, Auyeung B, Holt RJ, Allison C, … Baron-Cohen S (2015). Enhancing the precision of our understanding about mentalizing in adults with autism. BioRxiv. 10.1101/034454

[R173] Loth E, Gómez JC, & Happé F (2008). Event schemas in autism spectrum disorders: The role of theory of mind and weak central coherence. Journal of Autism and Developmental Disorders, 38, 449–463. 10.1007/s10803-007-0412-2 17668309

[R174] Loukusa S, Mäkinen L, Kuusikko-Gauffin S, Ebeling H, & Moilanen I (2014). Theory of mind and emotion recognition skills in children with specific language impairment, autism spectrum disorder and typical development: Group differences and connection to knowledge of grammatical morphology, word-finding abilities and verbal working memory. International Journal of Language & Communication Disorders, 49, 498–507. 10.1111/1460-6984.12091 24888967

[R175] Lukito S, Jones CRG, Pickles A, Baird G, Happé F, Charman T, & Simonoff E (2017). Specificity of executive function and theory of mind performance in relation to attention-deficit/hyperactivity symptoms in autism spectrum disorders. Molecular Autism, 8, 60 10.1186/s13229-017-0177-1 29152165 PMC5680830

[R176] Lundy JEB (2002). Age and language skills of deaf children in relation to theory of mind development. Journal of Deaf Studies and Deaf Education, 7, 41–56. 10.1093/deafed/7.1.41 15451885

[R177] Lunn J, Lewis C, & Sherlock C (2015). Impaired performance on advanced theory of mind tasks in children with epilepsy is related to poor communication and increased attention problems. Epilepsy & Behavior, 43, 109–116. 10.1016/j.yebeh.2014.11.010 25601584

[R178] Mash EJ, & Wolfe DA (2015). Abnormal child psychology (6th ed.). Belmont, CA: Wadsworth.

[R179] Mayes LC, Cohen DJ, & Klin A (1993). Experiencing self and others: A psychoanalytic perspective on theory of mind and autism In Baron-Cohen S, Tager-Flusberg H, & Cohen DJ (Eds.), Understanding other minds: Perspectives from autism (pp. 450–465). Oxford, United Kingdom: Oxford University Press.

[R180] McAleer P, Kay JW, Pollick FE, & Rutherford MD (2011). Intention perception in high functioning people with autism spectrum disorders using animacy displays derived from human actions. Journal of Autism and Developmental Disorders, 41, 1053–1063. 10.1007/s10803-010-1130-8 21069445

[R181] McCleery A, Divilbiss M, St-Hilaire A, Aakre JM, Seghers JP, Bell EK, & Docherty NM (2012). Predicting social functioning in schizotypy: An investigation of the relative contributions of theory of mind and mood. Journal of Nervous and Mental Disease, 200, 147–152. 22297312 10.1097/NMD.0b013e3182439533 PMC4431998

[R182] Melchers M, Montag C, Markett S, & Reuter M (2015). Assessment of empathy via self-report and behavioural paradigms: Data on convergent and discriminant validity. Cognitive Neuropsychiatry, 20, 157–171. 10.1080/13546805.2014.991781 25530230

[R183] Meristo M, Falkman KW, Hjelmquist E, Tedoldi M, Surian L, & Siegal M (2007). Language access and theory of mind reasoning: Evidence from deaf children in bilingual and oralist environments. Developmental Psychology, 43, 1156–1169. 10.1037/0012-1649.43.5.1156 17723042

[R184] Milligan K, Astington JW, & Dack LA (2007). Language and theory of mind: Meta-analysis of the relation between language ability and false-belief understanding. Child Development, 78, 622–646. 10.1111/j.1467-8624.2007.01018.x 17381794

[R185] Milton DEM (2012). On the ontological status of autism: The “double empathy problem.” Disability & Society, 27, 883–887. 10.1080/09687599.2012.710008

[R186] Minter ME, Hobson RP, & Bishop M (1998). Congenital visual impairment and “theory of mind.” British Journal of Developmental Psychology, 16, 183–196. 10.1111/j.2044-835X.1998.tb00918.x

[R187] Mitchell P (1997). Introduction to theory of mind: Children, autism and apes. London, United Kingdom: Arnold.

[R188] Moeller MP, & Schick B (2006). Relations between maternal input and theory of mind understanding in deaf children. Child Development, 77, 751–766. 10.1111/j.1467-8624.2006.00901.x 16686799

[R189] Moran JM, Young LL, Saxe R, Lee SM, O’Young D, Mavros PL, & Gabrieli JD (2011). Impaired theory of mind for moral judgment in high-functioning autism. Proceedings of the National Academy of Sciences of the United States of America, 108, 2688–2692. 10.1073/pnas.1011734108 21282628 PMC3041087

[R190] Muller F, Simion A, Reviriego E, Galera C, Mazaux JM, Barat M, & Joseph PA (2010). Exploring theory of mind after severe traumatic brain injury. Cortex, 46, 1088–1099. 10.1016/j.cortex.2009.08.014 19828142

[R191] Murray K, Johnston K, Cunnane H, Kerr C, Spain D, Gillan N, … Happé F (2017). A new test of advanced theory of mind: The “Strange Stories Film Task” captures social processing differences in adults with autism spectrum disorders. Autism Research, 10, 1120–1132. 10.1002/aur.1744 28296216

[R192] Myers DG (2009). Exploring psychology (8th ed.). New York, NY: Worth Publishers.

[R193] Myers DG (2012). Myers’ psychology for AP. New York, NY: Worth Publishers.

[R194] New Jersey v. Burr, 921 A. 2d 1135 (N.J. 2007)

[R195] Nicholson N (2013, 5 24). The empathy question: Theory of mind, culture, and understanding. Retrieved from http://www.thinkingautismguide.com/2013/05/the-empathy-question-theory-of-mind.html

[R196] Norbury CF (2005). The relationship between theory of mind and metaphor: Evidence from children with language impairment and autistic spectrum disorder. British Journal of Developmental Psychology, 23, 383–399. 10.1348/026151005X26732

[R197] Olderbak S, Wilhelm O, Olaru G, Geiger M, Brenneman MW, & Roberts RD (2015). A psychometric analysis of the reading the mind in the eyes test: Toward a brief form for research and applied settings. Frontiers in Psychology, 6, 1503 10.3389/fpsyg.2015.01503 26500578 PMC4593947

[R198] Oswald DP, & Ollendick TH (1989). Role taking and social competence in autism and mental retardation. Journal of Autism and Developmental Disorders, 19, 119–127. 10.1007/BF02212723 2708295

[R199] Ozonoff S, & McEvoy RE (1994). A longitudinal study of executive function and theory of mind development in autism. Development and Psychopathology, 6, 415–431. 10.1017/S0954579400006027

[R200] Ozonoff S, Pennington BF, & Rogers SJ (1991). Executive function deficits in high-functioning autistic individuals: Relationship to theory of mind. Journal of Child Psychology and Psychiatry, and Allied Disciplines, 32, 1081–1105. 10.1111/j.1469-7610.1991.tb00351.x 1787138

[R201] Ozonoff S, Rogers SJ, & Pennington BF (1991). Asperger’s syndrome: Evidence of an empirical distinction from high-functioning autism. Journal of Child Psychology and Psychiatry, and Allied Disciplines, 32, 1107–1122. 10.1111/j.1469-7610.1991.tb00352.x 1787139

[R202] Pantelis PC, & Kennedy DP (2017). Autism does not limit strategic thinking in the “beauty contest” game. Cognition, 160, 91–97. 10.1016/j.cognition.2016.12.015 28081516 PMC5303671

[R203] Payne JM, Porter M, Pride NA, & North KN (2016). Theory of mind in children with Neurofibromatosis Type 1. Neuropsychology, 30, 439–448. 10.1037/neu0000262 26752121

[R204] Peterson CC (2000). Kindred spirits: Influences of siblings’ perspectives on theory of mind. Cognitive Development, 15, 435–455. 10.1016/S0885-2014(01)00040-5

[R205] Peterson C (2014). Theory of mind understanding and empathic behavior in children with autism spectrum disorders. International Journal of Developmental Neuroscience, 39, 16–21. 10.1016/j.ijdevneu.2014.05.002 24875777

[R206] Peterson CC, Peterson JL, & Webb J (2000). Factors influencing the development of a theory of mind in blind children. British Journal of Developmental Psychology, 18, 431–447. 10.1348/026151000165788

[R207] Peterson CC, & Siegal M (1995). Deafness, conversation and theory of mind. Journal of Child Psychology and Psychiatry, and Allied Disciplines, 36, 459–474. 10.1111/j.1469-7610.1995.tb01303.x 7782409

[R208] Peterson CC, Slaughter V, & Wellman HM (2018). Nimble negotiators: How theory of mind (ToM) interconnects with persuasion skills in children with and without ToM delay. Developmental Psychology, 54, 494–509. 10.1037/dev0000451 29154648

[R209] Peterson CC, Wellman HM, & Slaughter V (2012). The mind behind the message: Advancing theory-of-mind scales for typically developing children, and those with deafness, autism, or Asperger syndrome. Child Development, 83, 469–485. 10.1111/j.1467-8624.2011.01728.x 22304467 PMC3642852

[R210] Peterson E, & Miller SF (2012). The eyes test as a measure of individual differences: How much of the variance reflects verbal IQ? Frontiers in Psychology, 3, 220 10.3389/fpsyg.2012.00220 22783217 PMC3389807

[R211] Pinker S (2002). The blank slate: The modern denial of human nature. New York, NY: Penguin.

[R212] Pino MC, Mazza M, Mariano M, Peretti S, Dimitriou D, Masedu F, … Franco F (2017). Simple mindreading abilities predict complex theory of mind: Developmental delay in autism spectrum disorders. Journal of Autism and Developmental Disorders, 47, 2743–2756. 10.1007/s10803-017-3194-1 28597142

[R213] Ponnet K, Buysse A, Roeyers H, & De Corte K (2005). Empathic accuracy in adults with a pervasive developmental disorder during an unstructured conversation with a typically developing stranger. Journal of Autism and Developmental Disorders, 35, 585–600. 10.1007/s10803-005-0003-z 16167090

[R214] Ponnet KS, Roeyers H, Buysse A, De Clercq A, & Van der Heyden E (2004). Advanced mind-reading in adults with Asperger syndrome. Autism, 8, 249–266. 10.1177/1362361304045214 15358869

[R215] Prior M, Dahlstrom B, & Squires T-L (1990). Autistic children’s knowledge of thinking and feeling states in other people. Journal of Child Psychology and Psychiatry, and Allied Disciplines, 31, 587–601. 10.1111/j.1469-7610.1990.tb00799.x 2365761

[R216] Ragsdale G, & Foley RA (2011). A maternal influence on Reading the mind in the Eyes mediated by executive function: Differential parental influences on full and half-siblings. PLoS ONE, 6, e23236 10.1371/journal.pone.0023236 21850264 PMC3151289

[R217] Rajendran G, & Mitchell P (2007). Cognitive theories of autism. Developmental Review, 27, 224–260. 10.1016/j.dr.2007.02.001

[R218] Rasmussen C, Wyper K, & Talwar V (2009). The relation between theory of mind and executive functions in children with fetal alcohol spectrum disorders. The Canadian Journal of Clinical Pharmacology, 16, e370–e380. Retrieved from https://www.ncbi.nlm.nih.gov/pubmed/19638654 19638654

[R219] Raud T, Kaldoja ML, & Kolk A (2015). Relationship between social competence and neurocognitive performance in children with epilepsy. Epilepsy & Behavior, 52, 93–101. 10.1016/j.yebeh.2015.08.028 26409136

[R220] Realo A, Allik J, Nolvak A, Valk R, Ruus T, Schmidt M, & Eilola T (2003). Mind-reading ability: Beliefs and performance. Journal of Research in Personality, 37, 420–445. 10.1016/S0092-6566(03)00021-7

[R221] Reidy RE, Ross RG, & Hunter SK (2013). Theory of mind development is impaired in 4-year-old children with prenatal exposure to maternal tobacco smoking. International Neuropsychiatric Disease Journal, 1, 24–34. 10.9734/INDJ/2013/3916 25558458 PMC4280667

[R222] Rhys-Jones SL, & Ellis HD (2000). Theory of mind: Deaf and hearing children’s comprehension of picture stories and judgments of social situa tions. Journal of Deaf Studies and Deaf Education, 5, 248–265. 10.1093/deafed/5.3.248 15454504

[R223] Roeyers H, Buysse A, Ponnet K, & Pichal B (2001). Advancing advanced mind-reading tests: Empathic accuracy in adults with a pervasive developmental disorder. Journal of Child Psychology and Psychiatry, and Allied Disciplines, 42, 271–278. 10.1111/1469-7610.00718 11280423

[R224] Ronald A, Viding E, Happé F, & Plomin R (2006). Individual differences in theory of mind ability in middle childhood and links with verbal ability and autistic traits: A twin study. Social Neuroscience, 1, 412–425. 10.1080/17470910601068088 18633802

[R225] Russell J, & Hill EL (2001). Action-monitoring and intention reporting in children with autism. Journal of Child Psychology and Psychiatry, and Allied Disciplines, 42, 317–328. 10.1111/1469-7610.00725 11321201

[R226] Russell PA, Hosie JA, Gray CD, Scott C, Hunter N, Banks JS, & Macaulay MC (1998). The development of theory of mind in deaf children. Journal of Child Psychology and Psychiatry, and Allied Disciplines, 39, 903–910. 9758198

[R227] Rutherford MD, Baron-Cohen S, & Wheelwright S (2002). Reading the mind in the voice: A study with normal adults and adults with Asperger syndrome and high functioning autism. Journal of Autism and Developmental Disorders, 32, 189–194. 10.1023/A:1015497629971 12108620

[R228] Salter G, Seigal A, Claxton M, Lawrence K, & Skuse D (2008). Can autistic children read the mind of an animated triangle? Autism, 12, 349–371. 10.1177/1362361308091654 18579644

[R229] San José Cáceres A, Keren N, Booth R, & Happé F (2014). Assessing theory of mind nonverbally in those with intellectual disability and ASD: The penny hiding game. Autism Research, 7, 608–616. 10.1002/aur.1405 25258194

[R230] Savina I, & Beninger RJ (2007). Schizophrenic patients treated with clozapine or olanzapine perform better on theory of mind tasks than those treated with risperidone or typical antipsychotic medications. Schizophrenia Research, 94, 128–138. 10.1016/j.schres.2007.04.010 17560766

[R231] Scassellati B (2002). Theory of mind for a humanoid robot. Autonomous Robots, 12, 13–24. 10.1023/A:1013298507114

[R232] Schaafsma SM, Pfaff DW, Spunt RP, & Adolphs R (2015). Deconstructing and reconstructing theory of mind. Trends in Cognitive Sciences, 19, 65–72. 10.1016/j.tics.2014.11.007 25496670 PMC4314437

[R233] Scheeren AM, de Rosnay M, Koot HM, & Begeer S (2013). Rethinking theory of mind in high-functioning autism spectrum disorder. Journal of Child Psychology and Psychiatry, and Allied Disciplines, 54, 628–635. 10.1111/jcpp.12007 23072222

[R234] Scherzer P, Leveillé E, Achim A, Boisseau E, & Stip E (2012). A study of theory of mind in paranoid schizophrenia: A theory or many theories? Frontiers in Psychology, 3, 432 10.3389/fpsyg.2012.00432 23162496 PMC3497936

[R235] Schneider D, Slaughter VP, Bayliss AP, & Dux PE (2013). A temporally sustained implicit theory of mind deficit in autism spectrum disorders. Cognition, 129, 410–417. 10.1016/j.cognition.2013.08.004 23994318

[R236] Schuwerk T, Priewasser B, Sodian B, & Perner J (2018). The robustness and generalizability of findings on spontaneous false belief sensitivity: A replication attempt. Royal Society Open Science, 5, 72273 10.1098/rsos.172273 PMC5990829 29892412

[R237] Schuwerk T, Vuori M, & Sodian B (2015). Implicit and explicit theory of mind reasoning in autism spectrum disorders: The impact of experience. Autism, 19, 459–468. 10.1177/1362361314526004 24627427

[R238] Scottish Autism. (n.d.) Impaired theory of mind. Retrieved from http://www.scottishautism.org/about-autism/about-autism/thinking-styles/impaired-theory-mind

[R239] Seattle Children’s Hospital. (2016, 3 10). Autism and theory of mind [Blog post]. Retrieved from http://theautismblog.seattlechildrens.org/autism-theory-mind/

[R240] Sebanz N, Knoblich G, Stumpf L, & Prinz W (2005). Far from action-blind: Representation of others’ actions in individuals with autism. Cognitive Neuropsychology, 22, 433–454. 10.1080/02643290442000121 21038260

[R241] Senju A, Southgate V, White S, & Frith U (2009). Mindblind eyes: An absence of spontaneous theory of mind in Asperger syndrome. Science, 325, 883–885. 10.1126/science.1176170 19608858

[R242] Shahrivar Z, Tehrani-Doost M, Khorrami Banaraki A, Mohammadzadeh A, & Happé F (2017). Normative data and psychometric properties of a Farsi translation of the strange stories test. Autism Research, 10, 1960–1967. 10.1002/aur.1844 28801936

[R243] Shaked M, Gamliel I, & Yirmiya N (2006). Theory of mind abilities in young siblings of children with autism. Autism, 10, 173–187. 10.1177/1362361306062023 16613866

[R244] Sharp C, & Vanwoerden S (2014). Social cognition: Empirical contribution. The developmental building blocks of psychopathic traits: Revisiting the role of theory of mind. Journal of Personality Disorders, 28, 78–95. 10.1521/pedi.2014.28.1.78 24344889

[R245] Sigelman CK, & Rider EA (2017). Life-span human development (9th ed.). Boston, MA: Cengage Learning.

[R246] Simmons JP, Nelson LD, & Simonsohn U (2013, 1). Life after p-hacking. Paper presented at Society for Personality and Social Psychology, New Orleans, LA 10.2139/ssrn.2205186

[R247] Sinclair J (1993). Don’t mourn for us. Our Voice, 1, 5–6.

[R248] Singer T, & Fehr E (2005). The neuroeconomics of mind reading and empathy. The American Economic Review, 95, 340–345. 10.1257/000282805774670103 29125271

[R249] Sivaratnam CS, Cornish K, Gray KM, Howlin P, & Rinehart NJ (2012). Brief report: Assessment of the social-emotional profile in children with autism spectrum disorders using a novel comic strip task. Journal of Autism and Development Disorders, 42, 2505–2512. 10.1007/s10803-012-1498-8 22419380

[R250] Smith DL (2007). The most dangerous animal: Human nature and the origins of war. New York, NY: St. Martin’s Press.

[R251] Solomon M, Goodlin-Jones BL, & Anders TF (2004). A social adjustment enhancement intervention for high functioning autism, Asperger’s syndrome, and pervasive developmental disorder NOS. Journal of Autism and Developmental Disorders, 34, 649–668. 10.1007/s10803-004-5286-y 15679185

[R252] Soper HV, & Murray MO (2012). Autism In Noggle CA, Dean RS, & Horton AM (Eds.), The Encyclopedia of neuropsychological disorders (pp. 125–128). New York, NY: Springer Publishing.

[R253] Sparrevohn R, & Howie PM (1995). Theory of mind in children with autistic disorder: Evidence of developmental progression and the role of verbal ability. Journal of Child Psychology and Psychiatry, and Allied Disciplines, 36, 249–263. 10.1111/j.1469-7610.1995.tb01823.x 7759589

[R254] Spek AA, Scholte EM, &Van Berckelaer-Onnes IA (2010). Theory of mind in adults with HFA and Asperger syndrome. Journal of Autism and Developmental Disorders, 40, 280–289. 10.1007/s10803-009-0860-y 19763808

[R255] Spellman BA (2015). A short (personal) future history of revolution 2.0. Perspectives on Psychological Science, 10, 886–899. 10.1177/1745691615609918 26581743

[R256] Stanford AD, Messinger J, Malaspina D, & Corcoran CM (2011). Theory of mind in patients at clinical high risk for psychosis. Schizophrenia Research, 131, 11–17. 10.1016/j.schres.2011.06.005 21757324 PMC3159813

[R257] Steele S, Joseph RM, & Tager-Flusberg H (2003). Brief report: Developmental change in theory of mind abilities in children with autism. Journal of Autism and Developmental Disorders, 33, 461–467. 10.1023/A:1025075115100 12959426

[R258] Suddendorf T, & Corballis MC (1997). Mental time travel and the evolution of the human mind. Genetic, Social, and General Psychology Monographs, 123, 133–167. 9204544

[R259] Tackett JL, Lilienfeld SO, Patrick CJ, Johnson SL, Krueger RF, Miller JD, … Shrout PE (2017). It’s time to broaden the replicability conversation: Thoughts for and from clinical psychological science. Perspectives on Psychological Science, 12, 742–756. 10.1177/1745691617690042 28972844

[R260] Tager-Flusberg H (2001). A reexamination of the theory of mind hypothesis of autism In Burack JA, Charman T, Yirmiya N, & Zelazo PR (Eds.), The development of autism: Perspectives from theory and research (pp. 173–193). Mawhah, NJ: Erlbaum.

[R261] Tager-Flusberg H (2007). Evaluating the theory-of-mind hypothesis of autism. Current Directions in Psychological Science, 16, 311–315. 10.1111/j.1467-8721.2007.00527.x

[R262] Tager-Flusberg H, & Sullivan K (1994). A second look at second-order belief attribution in autism. Journal of Autism and Developmental Disorders, 24, 577–586. 10.1007/BF02172139 7814307

[R263] Travis L, Sigman M, & Ruskin E (2001). Links between social understanding and social behavior in verbally able children with autism. Journal of Autism and Developmental Disorders, 31, 119–130. 10.1023/A:1010705912731 11450811

[R264] Tsang T, Gillespie-Lynch K, & Hutman T (2016). Theory of mind indexes the broader autism phenotype in siblings of children with autism at school age. Autism Research and Treatment, 2016, 6309189 10.1155/2016/6309189 26881074 PMC4736958

[R265] Tso IF, Grove TB, & Taylor SF (2010). Emotional experience predicts social adjustment independent of neurocognition and social cognition in schizophrenia. Schizophrenia Research, 122, 156–163. 10.1016/j.schres.2009.12.007 20051314 PMC2891306

[R266] United States v. Geanakos, No. 2:13-cr-0404-GEB (E. D. Cal. 2017).

[R267] Valla JM, Ganzel BL, Yoder KJ, Chen GM, Lyman LT, Sidari AP, … Belmonte MK (2010). More than maths and mindreading: Sex differences in empathizing/systemizing covariance. Autism Research, 3, 174–184. 10.1002/aur.143 20589713

[R268] Van Herwegen J, Dimitriou D, & Rundblad G (2013). Performance on verbal and low-verbal false belief tasks: Evidence from children with Williams syndrome. Journal of Communication Disorders, 46, 440–448. 10.1016/j.jcomdis.2013.10.002 24239484

[R269] Vellante M, Baron-Cohen S, Melis M, Marrone M, Petretto DR, Masala C, & Preti A (2013). The “Reading the Mind in the Eyes” test: Systematic review of psychometric properties and a validation study in Italy. Cognitive Neuropsychiatry, 18, 326–354. 10.1080/13546805.2012.721728 23106125 PMC6345369

[R270] Vetter NC, Leipold K, Kliegel M, Phillips LH, & Altgassen M (2013). Ongoing development of social cognition in adolescence. Child Neuropsychology, 19, 615–629. 10.1080/09297049.2012.718324 22934659

[R271] VisualVox. (2017, 8 14). Without #TheoryOfMind, #ToSiriWithLove wouldn’t be the dumpster fire it is [Blog post]. Retrieved from http://visualvox.wordpress.com/2017/12/14/without-theoryofmind-tosiriwithlove-wouldnt-be-the-dumpster-fire-it-is/

[R272] Vivanti G, McCormick C, Young GS, Abucayan F, Hatt N, Nadig A, … Rogers SJ (2011). Intact and impaired mechanisms of action understanding in autism. Developmental Psychology, 47, 841–856. 10.1037/a0023105 21401220 PMC4922484

[R273] Voracek M, & Dressler SG (2006). Lack of correlation between digit ratio (2D:4D) and Baron-Cohen’s “Reading the Mind in the Eyes” test, empathy, systemising, and autism-spectrum quotients in a general population sample. Personality and Individual Differences, 41, 1481–1491. 10.1016/j.paid.2006.06.009

[R274] Watson AC, Nixon CL, Wilson A, & Capage L (1999). Social interaction skills and theory of mind in young children. Developmental Psychology, 35, 386–391. 10.1037/0012-1649.35.2.386 10082009

[R275] White SJ, Coniston D, Rogers R, & Frith U (2011). Developing the Frith-Happé animations: A quick and objective test of theory of mind for adults with autism. Autism Research, 4, 149–154. 10.1002/aur.174 21480540

[R276] White S, Hill E, Happé F, & Frith U (2009). Revisiting the strange stories: Revealing mentalizing impairments in autism. Child Development, 80, 1097–1117. 10.1111/j.1467-8624.2009.01319.x 19630896

[R277] Willey A, Subramaniam B, Hamilton JA, & Couperus J (2015). The mating life of geeks: Love, neuroscience, and the new autistic subject. Signs: Journal of Women in Culture and Society, 40, 369–391. 10.1086/678146

[R278] Wilson CE, Happé F, Wheelwright SJ, Ecker C, Lombardo MV, Johnston P, … the MRC AIMS Consortium. (2014). The neuropsychology of male adults with high-functioning autism or Asperger syndrome. Autism Research, 7, 568–581. 10.1002/aur.1394 24903974 PMC4489335

[R279] Yegorova M (2017, October 23). “The Good Doctor” sensationalizes certain aspects of autism. The Ticker. Retrieved from https://theticker.org/tickerarchive/the-good-doctor-sensationalizes-certain-aspects-of-autism

[R280] Yergeau M (2013). Clinically significant disturbance: On theorists who theorize theory of mind. Disability Studies Quarterly, 33, 4 10.18061/dsq.v33i4.3876

[R281] Yergeau M (2018). Authoring autism: On rhetoric and neurological queerness. Durham, NC: Duke University Press.

[R282] Yergeau M, & Huebner B (2017). Minding theory of mind. Journal of Social Philosophy, 48, 273–296. 10.1111/josp.12191

[R283] Yirmiya N, Erel O, Shaked M, & Solomonica-Levi D (1998). Meta-analyses comparing theory of mind abilities of individuals with autism, individuals with mental retardation, and normally developing individuals. Psychological Bulletin, 124, 283–307. 10.1037/0033-2909.124.3.283 9849110

[R284] Yirmiya N, & Shulman C (1996). Seriation, conservation, and theory of mind abilities in individuals with autism, individuals with mental retardation, and normally developing children. Child Development, 67, 2045–2059. 10.2307/1131608 9022228

[R285] Yirmiya N, Solomonica-Levi D, Shulman C, & Pilowsky T (1996). Theory of mind abilities in individuals with autism, Down syndrome, and mental retardation of unknown etiology: The role of age and intelligence. Journal of Child Psychology and Psychiatry, and Allied Disciplines, 37, 1003–1014. 10.1111/j.1469-7610.1996.tb01497.x 9119934

[R286] Zaki J, Bolger N, & Ochsner K (2008). It takes two: The interpersonal nature of empathic accuracy. Psychological Science, 19, 399–404. 10.1111/j.1467-9280.2008.02099.x 18399894

[R287] Zelazo PD, Burack JA, Benedetto E, & Frye D (1996). Theory of mind and rule use in individuals with Down’s syndrome: A test of the uniqueness and specificity claims. Journal of Child Psychology and Psychiatry, and Allied Disciplines, 37, 479–484. 10.1111/j.1469-7610.1996.tb01429.x 8735448

[R288] Zelazo PD, Jacques S, Burack JA, & Frye D (2002). The relation between theory of mind and rule use: Evidence from persons with autism-spectrum disorders. Infant and Child Development, 11, 171–195. 10.1002/icd.304

[R289] Zunshine L (2008). Theory of mind and fictions of embodied transparency. Narrative, 16, 65–92. 10.1353/nar.2008.0004

